# Supplementary material for: Genetic characteristics of human parainfluenza viruses 1–4 associated with acute lower respiratory tract infection in Chinese children, during 2015–2021
Source: Microbiol Spectr. 2024 Sep 12;12(10):e03432-23. doi: 10.1128/spectrum.03432-23 (PMC11448424; doi:10.1128/spectrum.03432-23)
Supplement: Figure S1 — ML trees of HN of HPIV1-4. [file spectrum.03432-23-s0001.pdf]

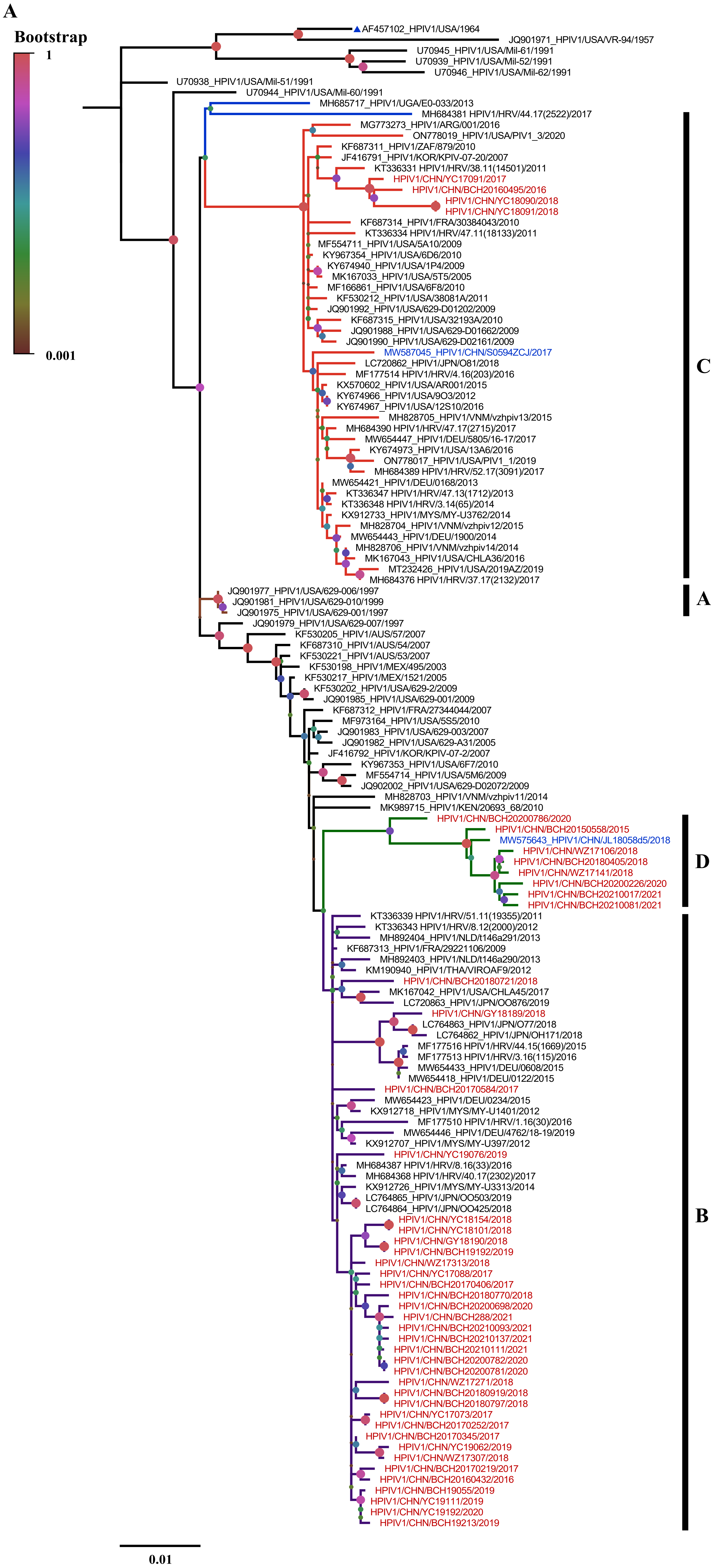
A
Bootstrap
1
0.001
AF457102_HPIV1/USA/1964
JQ901971_HPIV1/USA/VR-94/1957
U70945_HPIV1/USA/Mil-61/1991
U70939_HPIV1/USA/Mil-52/1991
U70946_HPIV1/USA/Mil-62/1991
U70938_HPIV1/USA/Mil-51/1991
U70944_HPIV1/USA/Mil-60/1991
MH685717_HPIV1/UGA/E0-033/2013
MH684381 HPIV1/HRV/44.17(2522)/2017
MG773273_HPIV1/ARG/001/2016
ON778019_HPIV1/USA/PIV1_3/2020
KF687311_HPIV1/ZAF/879/2010
JF416791_HPIV1/KOR/KPIV-07-20/2007
KT336331 HPIV1/HRV/38.11(14501)/2011
HPIV1/CHN/YC17091/2017
HPIV1/CHN/BCH20160495/2016
HPIV1/CHN/YC18090/2018
HPIV1/CHN/YC18091/2018
KF687314_HPIV1/FRA/30384043/2010
KT336334 HPIV1/HRV/47.11(18133)/2011
MF554711_HPIV1/USA/5A10/2009
KY967354_HPIV1/USA/6D6/2010
KY674940_HPIV1/USA/1P4/2009
MK167033_HPIV1/USA/5T5/2005
MF166861_HPIV1/USA/6F8/2010
KF530212_HPIV1/USA/38081A/2011
JQ901992_HPIV1/USA/629-D01202/2009
KF687315_HPIV1/USA/32193A/2010
JQ901988_HPIV1/USA/629-D01662/2009
JQ901990_HPIV1/USA/629-D02161/2009
MW587045_HPIV1/CHN/S0594ZCJ/2017
LC720862_HPIV1/JPN/O81/2018
MF177514 HPIV1/HRV/4.16(203)/2016
KX570602_HPIV1/USA/AR001/2015
KY674966_HPIV1/USA/9O3/2012
KY674967_HPIV1/USA/12S10/2016
MH828705_HPIV1/VNM/vzhpiv13/2015
MH684390 HPIV1/HRV/47.17(2715)/2017
MW654447_HPIV1/DEU/5805/16-17/2017
KY674973_HPIV1/USA/13A6/2016
ON778017_HPIV1/USA/PIV1_1/2019
MH684389 HPIV1/HRV/52.17(3091)/2017
MW654421_HPIV1/DEU/0168/2013
KT336347 HPIV1/HRV/47.13(1712)/2013
KT336348 HPIV1/HRV/3.14(65)/2014
KX912733_HPIV1/MYS/MY-U3762/2014
MH828704_HPIV1/VNM/vzhpiv12/2015
MW654443_HPIV1/DEU/1900/2014
MH828706_HPIV1/VNM/vzhpiv14/2014
MK167043_HPIV1/USA/CHLA36/2016
MT232426_HPIV1/USA/2019AZ/2019
MH684376 HPIV1/HRV/37.17(2132)/2017
C
JQ901977_HPIV1/USA/629-006/1997
JQ901981_HPIV1/USA/629-010/1999
JQ901975_HPIV1/USA/629-001/1997
A
JQ901979_HPIV1/USA/629-007/1997
KF530205_HPIV1/AUS/57/2007
KF687310_HPIV1/AUS/54/2007
KF530221_HPIV1/AUS/53/2007
KF530198_HPIV1/MEX/495/2003
KF530217_HPIV1/MEX/1521/2005
KF530202_HPIV1/USA/629-2/2009
JQ901985_HPIV1/USA/629-001/2009
KF687312_HPIV1/FRA/27344044/2007
MF973164_HPIV1/USA/5S5/2010
JQ901983_HPIV1/USA/629-003/2007
JQ901982_HPIV1/USA/629-A31/2005
JF416792_HPIV1/KOR/KPIV-07-2/2007
KY967353_HPIV1/USA/6F7/2010
MF554714_HPIV1/USA/5M6/2009
JQ902002_HPIV1/USA/629-D02072/2009
MH828703_HPIV1/VNM/vzhpiv11/2014
MK989715_HPIV1/KEN/20693_68/2010
HPIV1/CHN/BCH20200786/2020
HPIV1/CHN/BCH20150558/2015
MW575643_HPIV1/CHN/JL18058d5/2018
HPIV1/CHN/WZ17106/2018
HPIV1/CHN/BCH20180405/2018
HPIV1/CHN/WZ17141/2018
HPIV1/CHN/BCH20200226/2020
HPIV1/CHN/BCH20210017/2021
HPIV1/CHN/BCH20210081/2021
D
KT336339 HPIV1/HRV/51.11(19355)/2011
KT336343 HPIV1/HRV/8.12(2000)/2012
MH892404_HPIV1/NLD/t146a291/2013
KF687313_HPIV1/FRA/29221106/2009
MH892403_HPIV1/NLD/t146a290/2013
KM190940_HPIV1/THA/VIROAF9/2012
HPIV1/CHN/BCH20180721/2018
MK167042_HPIV1/USA/CHLA45/2017
LC720863_HPIV1/JPN/OO876/2019
HPIV1/CHN/GY18189/2018
LC764863_HPIV1/JPN/O77/2018
LC764862_HPIV1/JPN/OH171/2018
MF177516 HPIV1/HRV/44.15(1669)/2015
MF177513 HPIV1/HRV/3.16(115)/2016
MW654433_HPIV1/DEU/0608/2015
MW654418_HPIV1/DEU/0122/2015
HPIV1/CHN/BCH20170584/2017
MW654423_HPIV1/DEU/0234/2015
KX912718_HPIV1/MYS/MY-U1401/2012
MF177510 HPIV1/HRV/1.16(30)/2016
MW654446_HPIV1/DEU/4762/18-19/2019
KX912707_HPIV1/MYS/MY-U397/2012
HPIV1/CHN/YC19076/2019
MH684387 HPIV1/HRV/8.16(33)/2016
MH684368 HPIV1/HRV/40.17(2302)/2017
KX912726_HPIV1/MYS/MY-U3313/2014
LC764865_HPIV1/JPN/OO503/2019
LC764864_HPIV1/JPN/OO425/2018
HPIV1/CHN/YC18154/2018
HPIV1/CHN/YC18101/2018
HPIV1/CHN/GY18190/2018
HPIV1/CHN/BCH19192/2019
HPIV1/CHN/WZ17313/2018
HPIV1/CHN/YC17088/2017
HPIV1/CHN/BCH20170406/2017
HPIV1/CHN/BCH20180770/2018
HPIV1/CHN/BCH20200698/2020
HPIV1/CHN/BCH288/2021
HPIV1/CHN/BCH20210093/2021
HPIV1/CHN/BCH20210137/2021
HPIV1/CHN/BCH20210111/2021
HPIV1/CHN/BCH20200782/2020
HPIV1/CHN/BCH20200781/2020
HPIV1/CHN/WZ17271/2018
HPIV1/CHN/BCH20180919/2018
HPIV1/CHN/BCH20180797/2018
HPIV1/CHN/YC17073/2017
HPIV1/CHN/BCH20170252/2017
HPIV1/CHN/BCH20170345/2017
HPIV1/CHN/YC19062/2019
HPIV1/CHN/WZ17307/2018
HPIV1/CHN/BCH20170219/2017
HPIV1/CHN/BCH20160432/2016
HPIV1/CHN/BCH19055/2019
HPIV1/CHN/YC19111/2019
HPIV1/CHN/YC19192/2020
HPIV1/CHN/BCH19213/2019
B
0.01

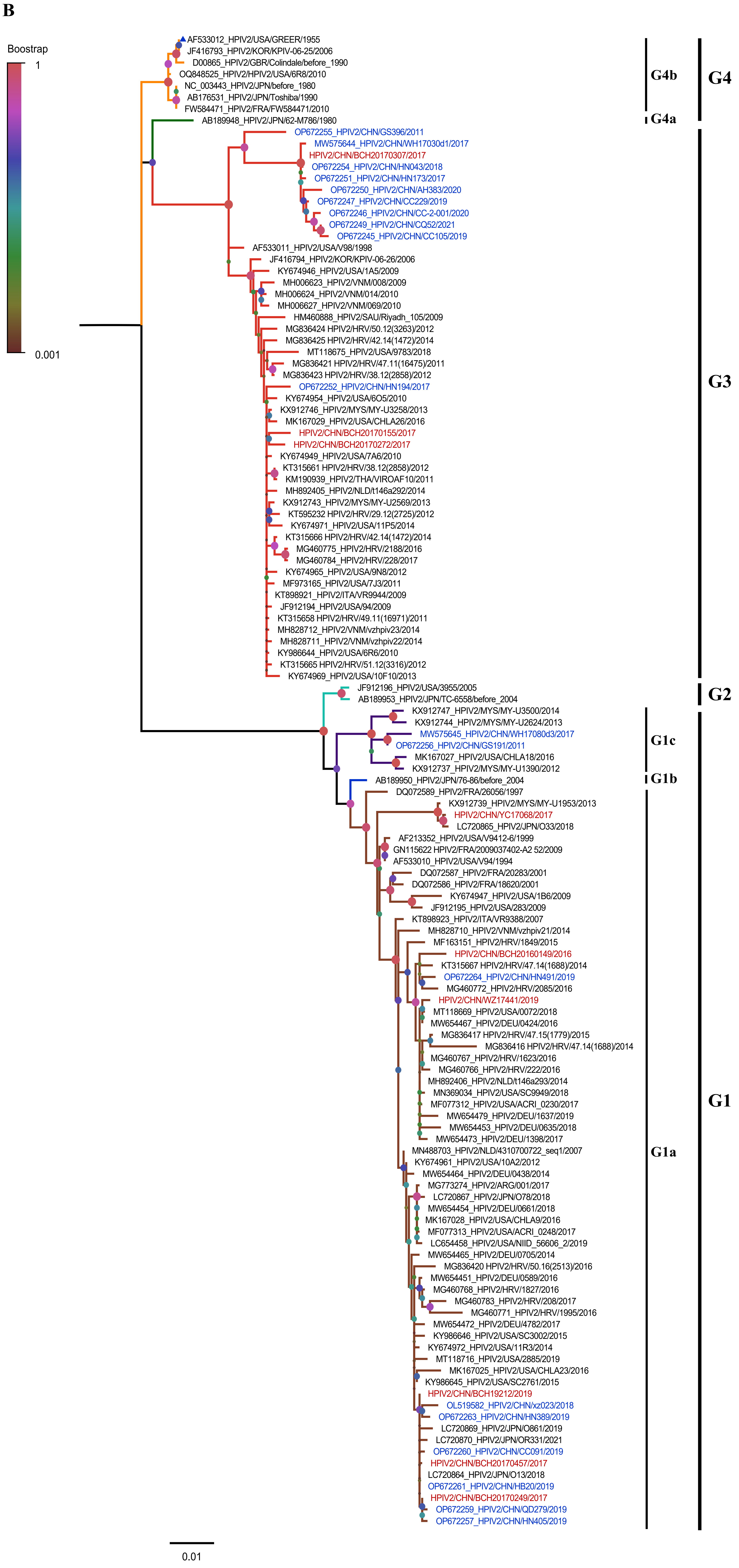

B
Boostrap
1
0.001
AF533012_HPIV2/USA/GREER/1955
JF416793_HPIV2/KOR/KPIV-06-25/2006
D00865_HPIV2/GBR/Colindale/before_1990
OQ848525_HPIV2/HPIV2/USA/6R8/2010
NC_003443_HPIV2/JPN/before_1980
AB176531_HPIV2/JPN/Toshiba/1990
FW584471_HPIV2/FRA/FW584471/2010
AB189948_HPIV2/JPN/62-M786/1980
OP672255_HPIV2/CHN/GS396/2011
MW575644_HPIV2/CHN/WH17030d1/2017
HPIV2/CHN/BCH20170307/2017
OP672254_HPIV2/CHN/HN043/2018
OP672251_HPIV2/CHN/HN173/2017
OP672250_HPIV2/CHN/AH383/2020
OP672247_HPIV2/CHN/CC229/2019
OP672246_HPIV2/CHN/CC-2-001/2020
OP672249_HPIV2/CHN/CQ52/2021
OP672245_HPIV2/CHN/CC105/2019
AF533011_HPIV2/USA/V98/1998
JF416794_HPIV2/KOR/KPIV-06-26/2006
KY674946_HPIV2/USA/1A5/2009
MH006623_HPIV2/VNM/008/2009
MH006624_HPIV2/VNM/014/2010
MH006627_HPIV2/VNM/069/2010
HM460888_HPIV2/SAU/Riyadh_105/2009
MG836424 HPIV2/HRV/50.12(3263)/2012
MG836425 HPIV2/HRV/42.14(1472)/2014
MT118675_HPIV2/USA/9783/2018
MG836421 HPIV2/HRV/47.11(16475)/2011
MG836423 HPIV2/HRV/38.12(2858)/2012
OP672252_HPIV2/CHN/HN194/2017
KY674954_HPIV2/USA/6O5/2010
KX912746_HPIV2/MYS/MY-U3258/2013
MK167029_HPIV2/USA/CHLA26/2016
HPIV2/CHN/BCH20170155/2017
HPIV2/CHN/BCH20170272/2017
KY674949_HPIV2/USA/7A6/2010
KT315661 HPIV2/HRV/38.12(2858)/2012
KM190939_HPIV2/THA/VIROAF10/2011
MH892405_HPIV2/NLD/t146a292/2014
KX912743_HPIV2/MYS/MY-U2569/2013
KT595232 HPIV2/HRV/29.12(2725)/2012
KY674971_HPIV2/USA/11P5/2014
KT315666 HPIV2/HRV/42.14(1472)/2014
MG460775_HPIV2/HRV/2188/2016
MG460784_HPIV2/HRV/228/2017
KY674965_HPIV2/USA/9N8/2012
MF973165_HPIV2/USA/7J3/2011
KT898921_HPIV2/ITA/VR9944/2009
JF912194_HPIV2/USA/94/2009
KT315658 HPIV2/HRV/49.11(16971)/2011
MH828712_HPIV2/VNM/vzhpiv23/2014
MH828711_HPIV2/VNM/vzhpiv22/2014
KY986644_HPIV2/USA/6R6/2010
KT315665 HPIV2/HRV/51.12(3316)/2012
KY674969_HPIV2/USA/10F10/2013
JF912196_HPIV2/USA/3955/2005
AB189953_HPIV2/JPN/TC-6558/before_2004
KX912747_HPIV2/MYS/MY-U3500/2014
KX912744_HPIV2/MYS/MY-U2624/2013
MW575645_HPIV2/CHN/WH17080d3/2017
OP672256_HPIV2/CHN/GS191/2011
MK167027_HPIV2/USA/CHLA18/2016
KX912737_HPIV2/MYS/MY-U1390/2012
AB189950_HPIV2/JPN/76-86/before_2004
DQ072589_HPIV2/FRA/26056/1997
KX912739_HPIV2/MYS/MY-U1953/2013
HPIV2/CHN/YC17068/2017
LC720865_HPIV2/JPN/O33/2018
AF213352_HPIV2/USA/V9412-6/1999
GN115622 HPIV2/FRA/2009037402-A2 52/2009
AF533010_HPIV2/USA/V94/1994
DQ072587_HPIV2/FRA/20283/2001
DQ072586_HPIV2/FRA/18620/2001
KY674947_HPIV2/USA/1B6/2009
JF912195_HPIV2/USA/283/2009
KT898923_HPIV2/ITA/VR9388/2007
MH828710_HPIV2/VNM/vzhpiv21/2014
MF163151_HPIV2/HRV/1849/2015
HPIV2/CHN/BCH20160149/2016
KT315667 HPIV2/HRV/47.14(1688)/2014
OP672264_HPIV2/CHN/HN491/2019
MG460772_HPIV2/HRV/2085/2016
HPIV2/CHN/WZ17441/2019
MT118669_HPIV2/USA/0072/2018
MW654467_HPIV2/DEU/0424/2016
MG836417 HPIV2/HRV/47.15(1779)/2015
MG836416 HPIV2/HRV/47.14(1688)/2014
MG460767_HPIV2/HRV/1623/2016
MG460766_HPIV2/HRV/222/2016
MH892406_HPIV2/NLD/t146a293/2014
MN369034_HPIV2/USA/SC9949/2018
MF077312_HPIV2/USA/ACRI_0230/2017
MW654479_HPIV2/DEU/1637/2019
MW654453_HPIV2/DEU/0635/2018
MW654473_HPIV2/DEU/1398/2017
MN488703_HPIV2/NLD/4310700722_seq1/2007
KY674961_HPIV2/USA/10A2/2012
MW654464_HPIV2/DEU/0438/2014
MG773274_HPIV2/ARG/001/2017
LC720867_HPIV2/JPN/O78/2018
MW654454_HPIV2/DEU/0661/2018
MK167028_HPIV2/USA/CHLA9/2016
MF077313_HPIV2/USA/ACRI_0248/2017
LC654458_HPIV2/USA/NIID_56606_2/2019
MW654465_HPIV2/DEU/0705/2014
MG836420 HPIV2/HRV/50.16(2513)/2016
MW654451_HPIV2/DEU/0589/2016
MG460768_HPIV2/HRV/1827/2016
MG460783_HPIV2/HRV/208/2017
MG460771_HPIV2/HRV/1995/2016
MW654472_HPIV2/DEU/4782/2017
KY986646_HPIV2/USA/SC3002/2015
KY674972_HPIV2/USA/11R3/2014
MT118716_HPIV2/USA/2885/2019
MK167025_HPIV2/USA/CHLA23/2016
KY986645_HPIV2/USA/SC2761/2015
HPIV2/CHN/BCH19212/2019
OL519582_HPIV2/CHN/xz023/2018
OP672263_HPIV2/CHN/HN389/2019
LC720869_HPIV2/JPN/O861/2019
LC720870_HPIV2/JPN/OR331/2021
OP672260_HPIV2/CHN/CC091/2019
HPIV2/CHN/BCH20170457/2017
LC720864_HPIV2/JPN/O13/2018
OP672261_HPIV2/CHN/HB20/2019
HPIV2/CHN/BCH20170249/2017
OP672259_HPIV2/CHN/QD279/2019
OP672257_HPIV2/CHN/HN405/2019
G4b
G4a
G4
G3
G2
G1c
G1b
G1a
G1
0.01

C

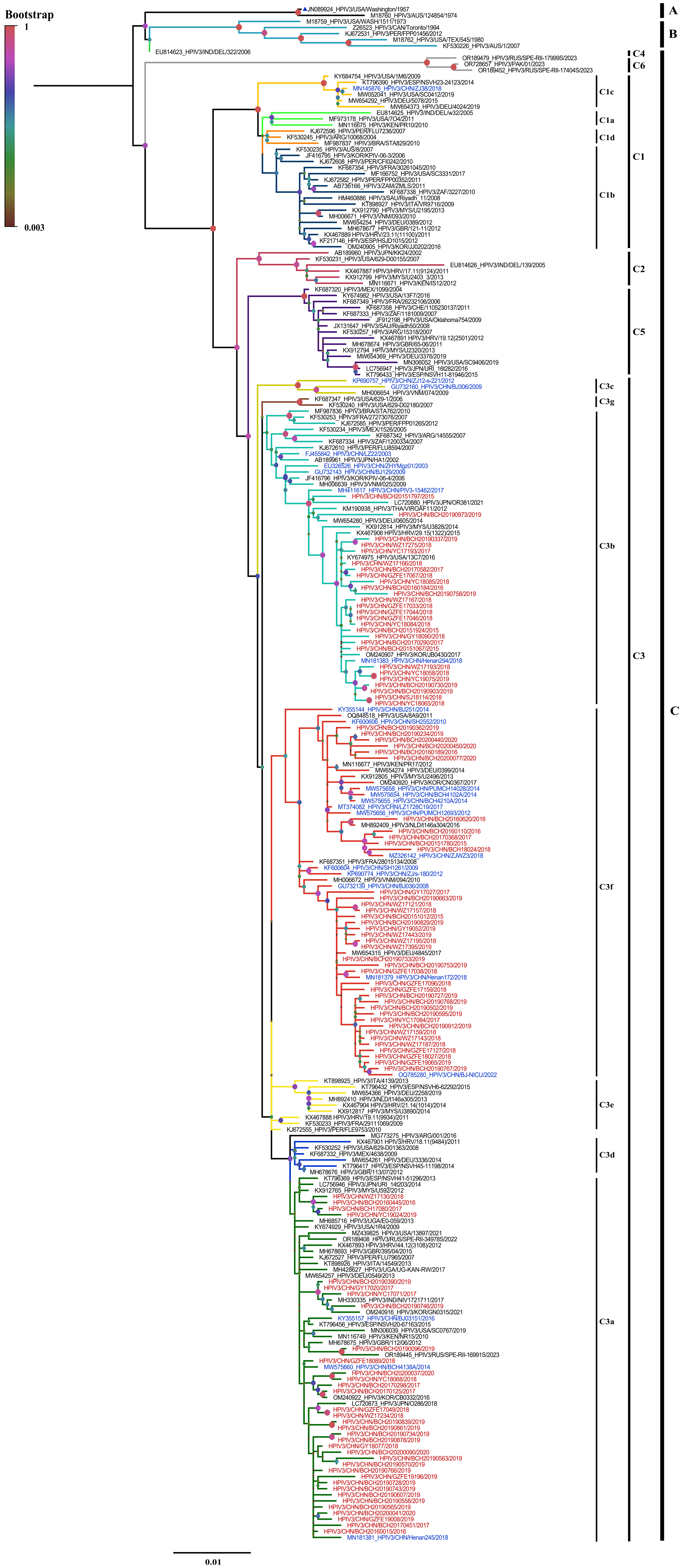

Bootstrap
1
0.003
A
B
C4
C6
C1c
C1a
C1d
C1
C1b
C2
C5
C3c
C3g
C3b
C3
C
C3f
C3e
C3d
C3a
0.01

D

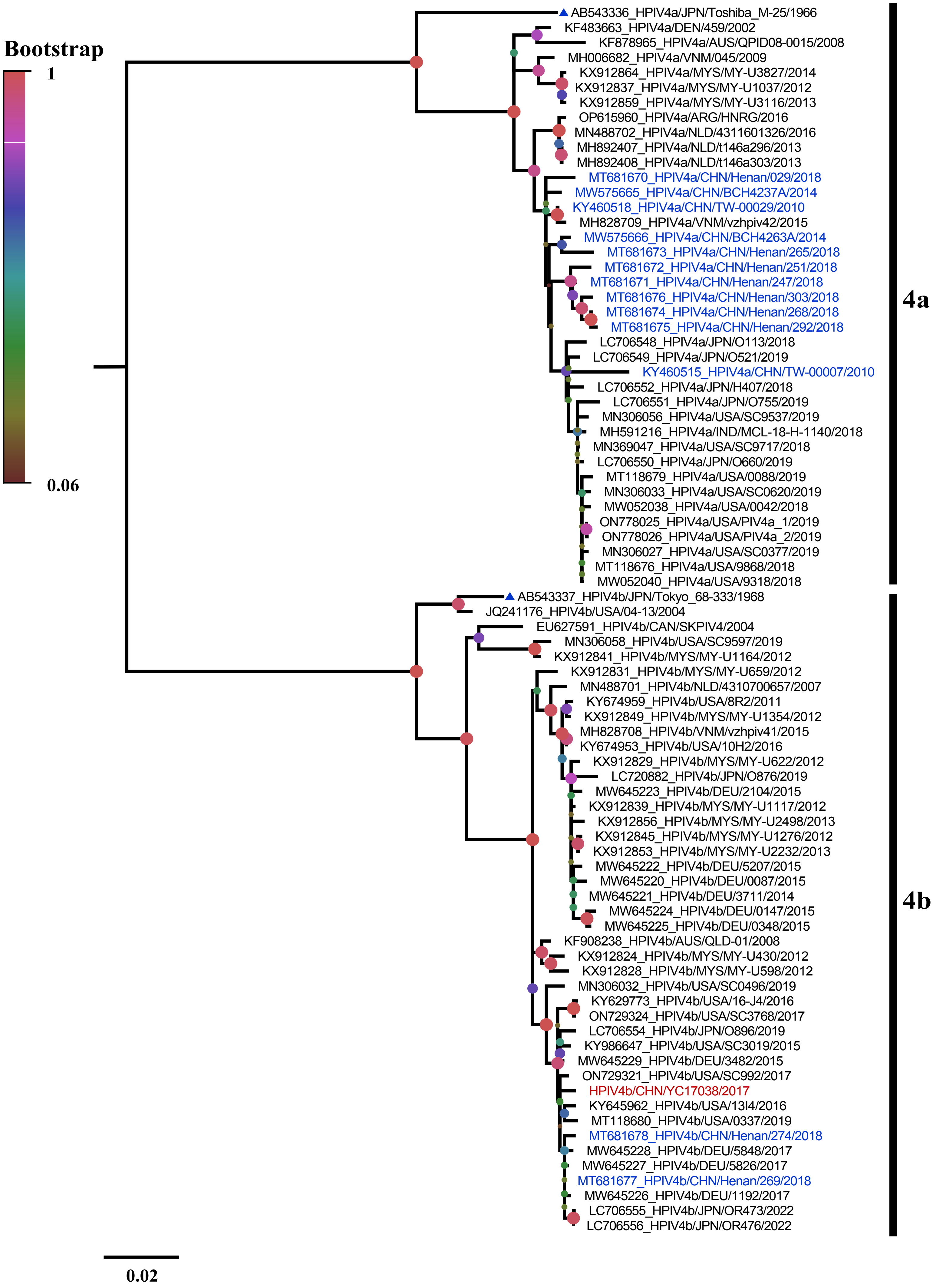

Supplementary figure 1. ML phylogenetic tree based on the full-length HN CDS sequence of HPIV1-4. Fig. (A–D) correspond to the trees of HPIV1, HPIV2, HPIV3, and HPIV4, respectively.The ML phylogenetic tree was constructed by the maximum likelihood method with 1000 bootstraps. The prototype strains, the strains obtained in this study and other Chinese strains are indicated by blue triangles, blue font, red font, respectively. The names of the strains include the GenBank number, serotype, country of isolation, name, and year of the collection. The country abbreviations ARG, AUS, BRA, CHE, CHN, DEU, ESP, FRA, GBR, HRV, ITA, IND, JPN, KEN, KOR, MEX, MYS, NLD, PER, RUS, SAU, THA, UGA, USA, VNM, ZAF and ZAM in the trees represent Argentina, Australia, Brazil, Switzerland, China, Germany, Spain, France, the United Kingdom, Croatia, Italy, India, Japan, Kenya, South Korea, Mexico, Malaysia, the Netherlands, Peru, Russia, Saudi Arabia, Thailand, the Republic of Uganda, the United States, Vietnam, the Republic of South Africa and the Republic of Zambia, respectively.
