# Supplementary material for: Genetic characteristics of human parainfluenza viruses 1–4 associated with acute lower respiratory tract infection in Chinese children, during 2015–2021
Source: Microbiol Spectr. 2024 Sep 12;12(10):e03432-23. doi: 10.1128/spectrum.03432-23 (PMC11448424; doi:10.1128/spectrum.03432-23)
Supplement: Figure S2 — ML trees of WGS of HPIV1-4. [file spectrum.03432-23-s0002.pdf]

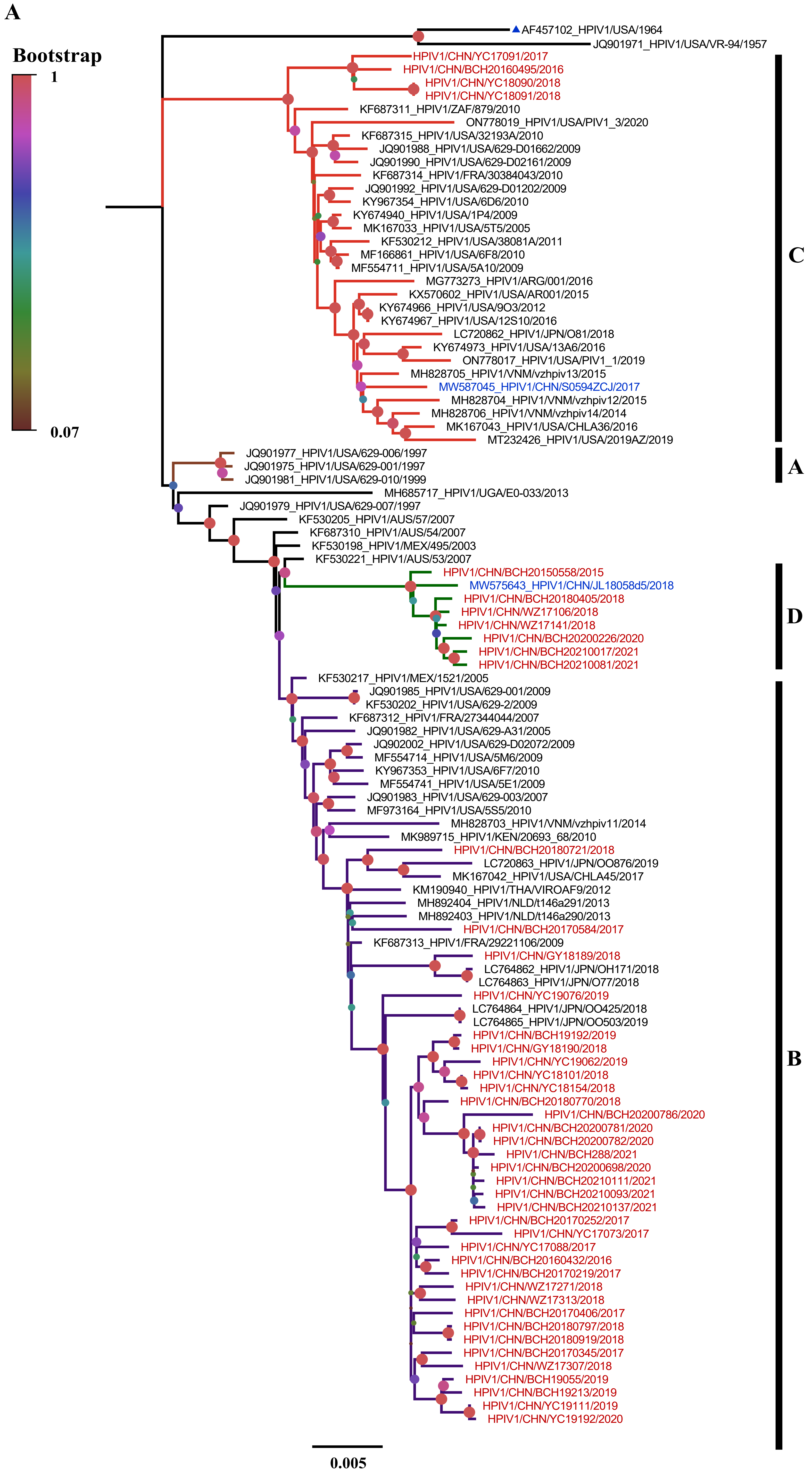
A
Bootstrap
1
0.07
AF457102_HPIV1/USA/1964
JQ901971_HPIV1/USA/VR-94/1957
HPIV1/CHN/YC17091/2017
HPIV1/CHN/BCH20160495/2016
HPIV1/CHN/YC18090/2018
HPIV1/CHN/YC18091/2018
KF687311_HPIV1/ZAF/879/2010
ON778019_HPIV1/USA/PIV1_3/2020
KF687315_HPIV1/USA/32193A/2010
JQ901988_HPIV1/USA/629-D01662/2009
JQ901990_HPIV1/USA/629-D02161/2009
KF687314_HPIV1/FRA/30384043/2010
JQ901992_HPIV1/USA/629-D01202/2009
KY967354_HPIV1/USA/6D6/2010
KY674940_HPIV1/USA/1P4/2009
MK167033_HPIV1/USA/5T5/2005
KF530212_HPIV1/USA/38081A/2011
MF166861_HPIV1/USA/6F8/2010
MF554711_HPIV1/USA/5A10/2009
MG773273_HPIV1/ARG/001/2016
KX570602_HPIV1/USA/AR001/2015
KY674966_HPIV1/USA/9O3/2012
KY674967_HPIV1/USA/12S10/2016
LC720862_HPIV1/JPN/O81/2018
KY674973_HPIV1/USA/13A6/2016
ON778017_HPIV1/USA/PIV1_1/2019
MH828705_HPIV1/VNM/vzhpiv13/2015
MW587045_HPIV1/CHN/S0594ZCJ/2017
MH828704_HPIV1/VNM/vzhpiv12/2015
MH828706_HPIV1/VNM/vzhpiv14/2014
MK167043_HPIV1/USA/CHLA36/2016
MT232426_HPIV1/USA/2019AZ/2019
C
JQ901977_HPIV1/USA/629-006/1997
JQ901975_HPIV1/USA/629-001/1997
JQ901981_HPIV1/USA/629-010/1999
A
MH685717_HPIV1/UGA/E0-033/2013
JQ901979_HPIV1/USA/629-007/1997
KF530205_HPIV1/AUS/57/2007
KF687310_HPIV1/AUS/54/2007
KF530198_HPIV1/MEX/495/2003
KF530221_HPIV1/AUS/53/2007
HPIV1/CHN/BCH20150558/2015
MW575643_HPIV1/CHN/JL18058d5/2018
HPIV1/CHN/BCH20180405/2018
HPIV1/CHN/WZ17106/2018
HPIV1/CHN/WZ17141/2018
HPIV1/CHN/BCH20200226/2020
HPIV1/CHN/BCH20210017/2021
HPIV1/CHN/BCH20210081/2021
D
KF530217_HPIV1/MEX/1521/2005
JQ901985_HPIV1/USA/629-001/2009
KF530202_HPIV1/USA/629-2/2009
KF687312_HPIV1/FRA/27344044/2007
JQ901982_HPIV1/USA/629-A31/2005
JQ902002_HPIV1/USA/629-D02072/2009
MF554714_HPIV1/USA/5M6/2009
KY967353_HPIV1/USA/6F7/2010
MF554741_HPIV1/USA/5E1/2009
JQ901983_HPIV1/USA/629-003/2007
MF973164_HPIV1/USA/5S5/2010
MH828703_HPIV1/VNM/vzhpiv11/2014
MK989715_HPIV1/KEN/20693_68/2010
HPIV1/CHN/BCH20180721/2018
LC720863_HPIV1/JPN/OO876/2019
MK167042_HPIV1/USA/CHLA45/2017
KM190940_HPIV1/THA/VIROAF9/2012
MH892404_HPIV1/NLD/t146a291/2013
MH892403_HPIV1/NLD/t146a290/2013
HPIV1/CHN/BCH20170584/2017
KF687313_HPIV1/FRA/29221106/2009
HPIV1/CHN/GY18189/2018
LC764862_HPIV1/JPN/OH171/2018
LC764863_HPIV1/JPN/O77/2018
HPIV1/CHN/YC19076/2019
LC764864_HPIV1/JPN/OO425/2018
LC764865_HPIV1/JPN/OO503/2019
HPIV1/CHN/BCH19192/2019
HPIV1/CHN/GY18190/2018
HPIV1/CHN/YC19062/2019
HPIV1/CHN/YC18101/2018
HPIV1/CHN/YC18154/2018
HPIV1/CHN/BCH20180770/2018
HPIV1/CHN/BCH20200786/2020
HPIV1/CHN/BCH20200781/2020
HPIV1/CHN/BCH20200782/2020
HPIV1/CHN/BCH288/2021
HPIV1/CHN/BCH20200698/2020
HPIV1/CHN/BCH20210111/2021
HPIV1/CHN/BCH20210093/2021
HPIV1/CHN/BCH20210137/2021
HPIV1/CHN/BCH20170252/2017
HPIV1/CHN/YC17073/2017
HPIV1/CHN/YC17088/2017
HPIV1/CHN/BCH20160432/2016
HPIV1/CHN/BCH20170219/2017
HPIV1/CHN/WZ17271/2018
HPIV1/CHN/WZ17313/2018
HPIV1/CHN/BCH20170406/2017
HPIV1/CHN/BCH20180797/2018
HPIV1/CHN/BCH20180919/2018
HPIV1/CHN/BCH20170345/2017
HPIV1/CHN/WZ17307/2018
HPIV1/CHN/BCH19055/2019
HPIV1/CHN/BCH19213/2019
HPIV1/CHN/YC19111/2019
HPIV1/CHN/YC19192/2020
B
0.005

B

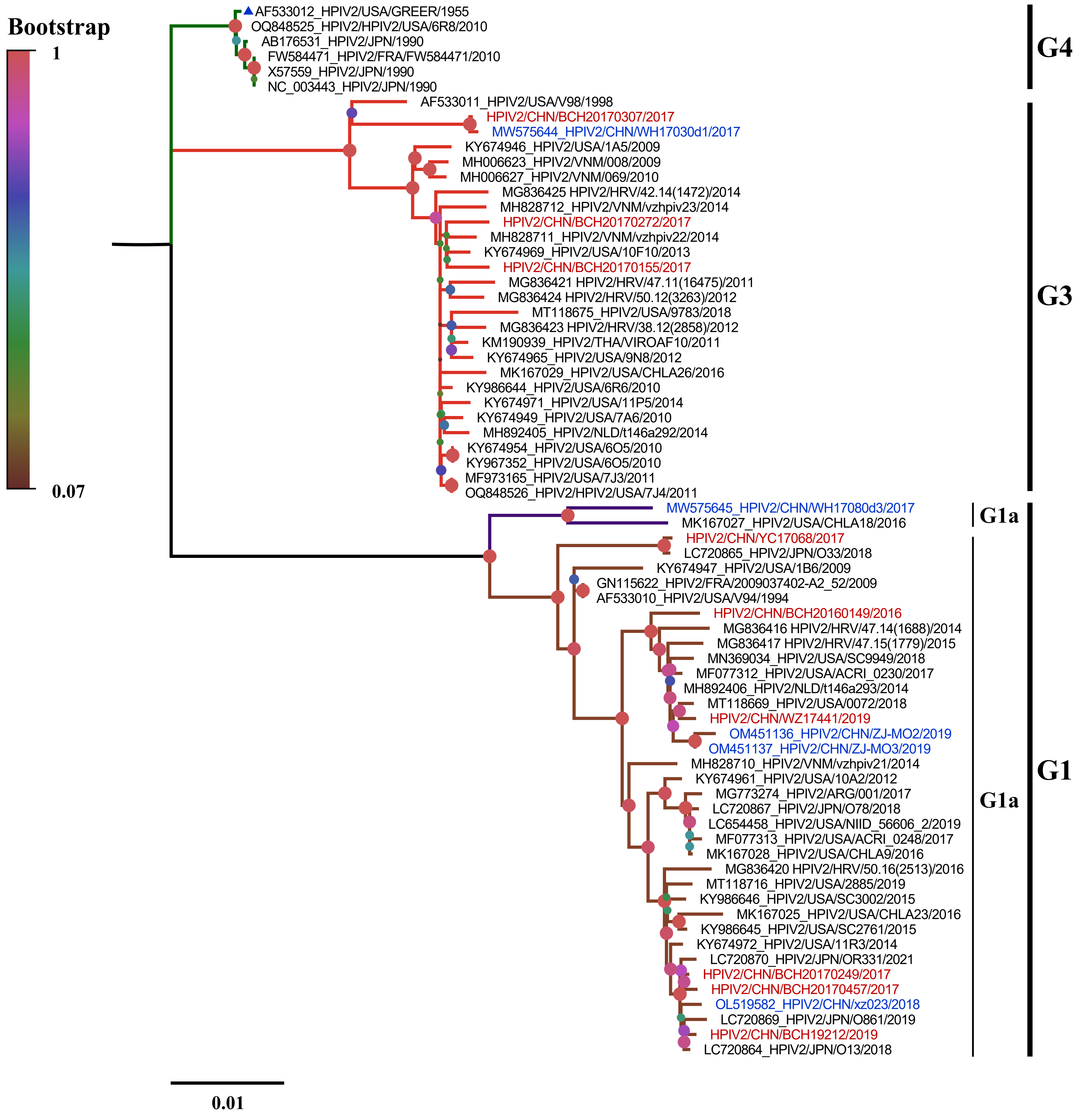
Bootstrap
1
0.07
AF533012_HPIV2/USA/GREER/1955
OQ848525_HPIV2/HPIV2/USA/6R8/2010
AB176531_HPIV2/JPN/1990
FW584471_HPIV2/FRA/FW584471/2010
X57559_HPIV2/JPN/1990
NC_003443_HPIV2/JPN/1990
AF533011_HPIV2/USA/V98/1998
HPIV2/CHN/BCH20170307/2017
MW575644_HPIV2/CHN/WH17030d1/2017
KY674946_HPIV2/USA/1A5/2009
MH006623_HPIV2/VNM/008/2009
MH006627_HPIV2/VNM/069/2010
MG836425 HPIV2/HRV/42.14(1472)/2014
MH828712_HPIV2/VNM/vzhpiv23/2014
HPIV2/CHN/BCH20170272/2017
MH828711_HPIV2/VNM/vzhpiv22/2014
KY674969_HPIV2/USA/10F10/2013
HPIV2/CHN/BCH20170155/2017
MG836421 HPIV2/HRV/47.11(16475)/2011
MG836424 HPIV2/HRV/50.12(3263)/2012
MT118675_HPIV2/USA/9783/2018
MG836423 HPIV2/HRV/38.12(2858)/2012
KM190939_HPIV2/THA/VIROAF10/2011
KY674965_HPIV2/USA/9N8/2012
MK167029_HPIV2/USA/CHLA26/2016
KY986644_HPIV2/USA/6R6/2010
KY674971_HPIV2/USA/11P5/2014
KY674949_HPIV2/USA/7A6/2010
MH892405_HPIV2/NLD/t146a292/2014
KY674954_HPIV2/USA/6O5/2010
KY967352_HPIV2/USA/6O5/2010
MF973165_HPIV2/USA/7J3/2011
OQ848526_HPIV2/HPIV2/USA/7J4/2011
MW575645_HPIV2/CHN/WH17080d3/2017
MK167027_HPIV2/USA/CHLA18/2016
HPIV2/CHN/YC17068/2017
LC720865_HPIV2/JPN/O33/2018
KY674947_HPIV2/USA/1B6/2009
GN115622_HPIV2/FRA/2009037402-A2_52/2009
AF533010_HPIV2/USA/V94/1994
HPIV2/CHN/BCH20160149/2016
MG836416 HPIV2/HRV/47.14(1688)/2014
MG836417 HPIV2/HRV/47.15(1779)/2015
MN369034_HPIV2/USA/SC9949/2018
MF077312_HPIV2/USA/ACRI_0230/2017
MH892406_HPIV2/NLD/t146a293/2014
MT118669_HPIV2/USA/0072/2018
HPIV2/CHN/WZ17441/2019
OM451136_HPIV2/CHN/ZJ-MO2/2019
OM451137_HPIV2/CHN/ZJ-MO3/2019
MH828710_HPIV2/VNM/vzhpiv21/2014
KY674961_HPIV2/USA/10A2/2012
MG773274_HPIV2/ARG/001/2017
LC720867_HPIV2/JPN/O78/2018
LC654458_HPIV2/USA/NIID_56606_2/2019
MF077313_HPIV2/USA/ACRI_0248/2017
MK167028_HPIV2/USA/CHLA9/2016
MG836420 HPIV2/HRV/50.16(2513)/2016
MT118716_HPIV2/USA/2885/2019
KY986646_HPIV2/USA/SC3002/2015
MK167025_HPIV2/USA/CHLA23/2016
KY986645_HPIV2/USA/SC2761/2015
KY674972_HPIV2/USA/11R3/2014
LC720870_HPIV2/JPN/OR331/2021
HPIV2/CHN/BCH20170249/2017
HPIV2/CHN/BCH20170457/2017
OL519582_HPIV2/CHN/xz023/2018
LC720869_HPIV2/JPN/O861/2019
HPIV2/CHN/BCH19212/2019
LC720864_HPIV2/JPN/O13/2018
G4
G3
G1a
G1
G1a
0.01

C

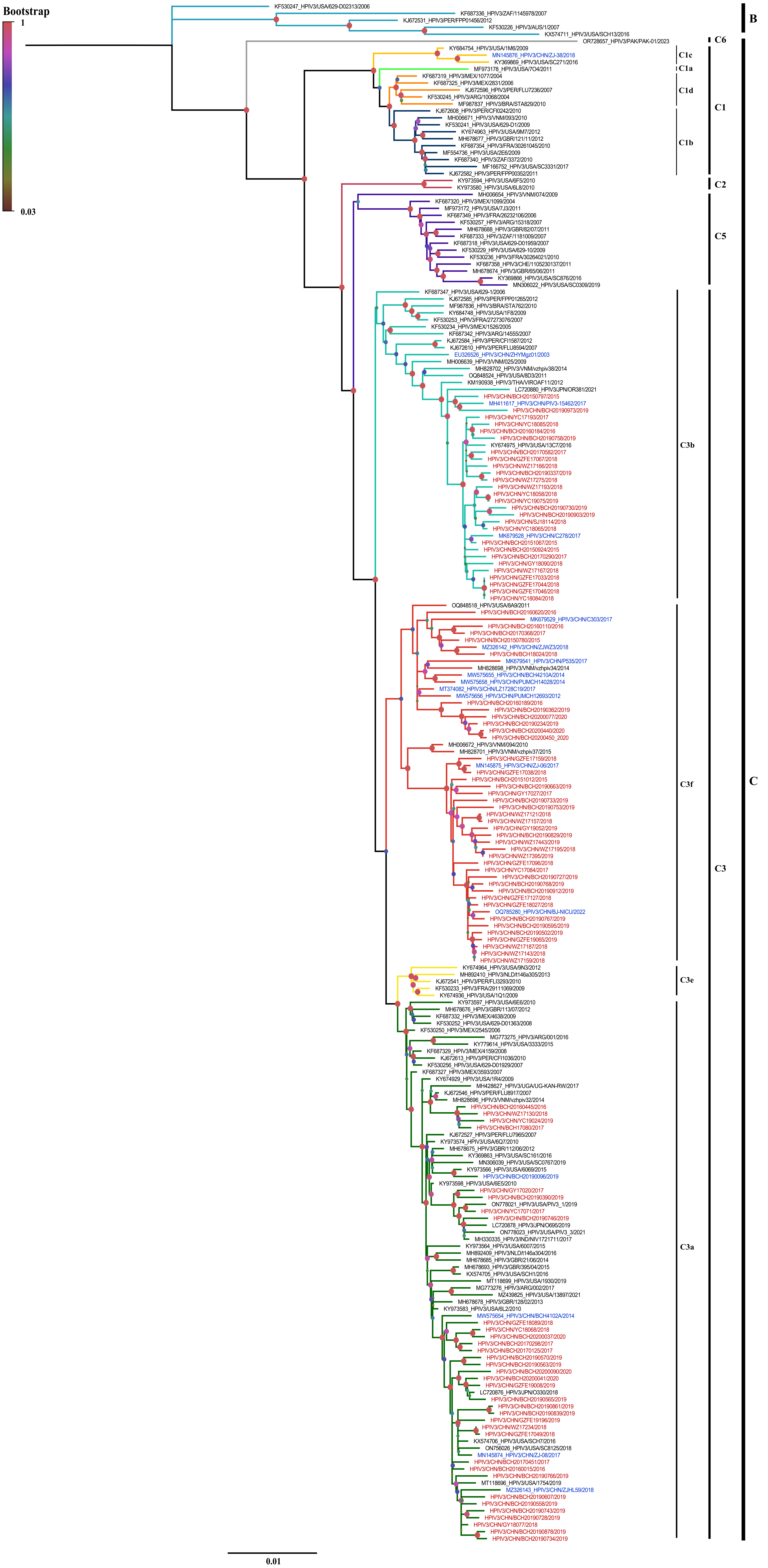
Bootstrap
1
0.03
0.01
B
C6
C1c
C1a
C1d
C1
C1b
C2
C5
C3b
C
C3f
C3
C3e
C3a

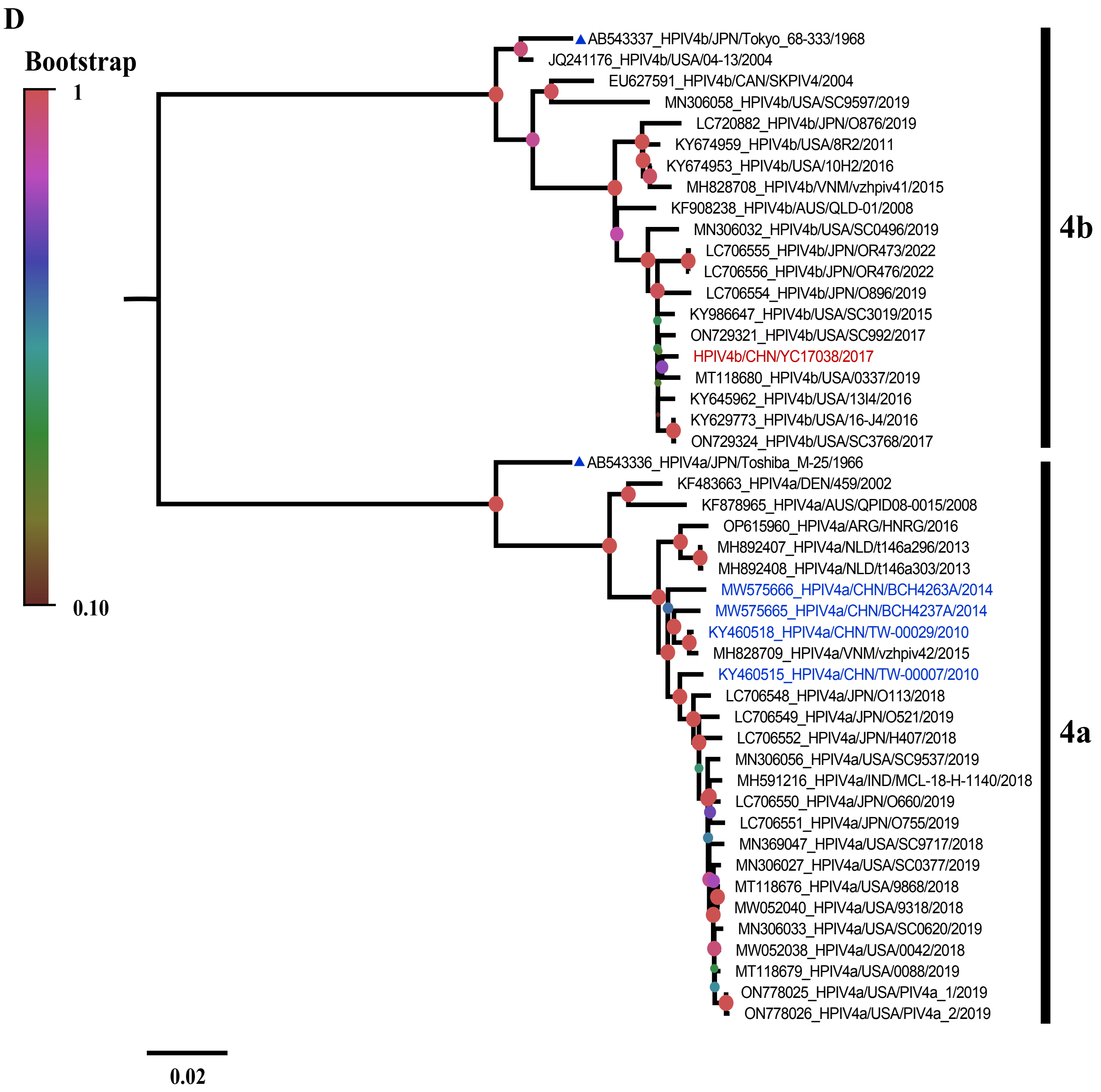

Supplementary figure 2. ML phylogenetic tree based on whole genome sequence of HPIV1-4. Fig. (A–D) correspond to the trees of HPIV1, HPIV2, HPIV3, and HPIV4, respectively. The ML phylogenetic tree was constructed by the maximum likelihood method with 1000 bootstraps. The prototype strains, the strains obtained in this study and other Chinese strains are indicated by blue triangles, blue font, red font, respectively. The names of the strains include the GenBank number, serotype, country of isolation, name, and year of the collection. The country abbreviations ARG, AUS, BRA, CHE, CHN, FRA, GBR, HRV, ITA, JPN, MEX, NLD, PER, THA, UGA, USA and VNM in the trees represent Argentina, Australia, Brazil, Switzerland, China, France, the United Kingdom, Croatia, Italy, Japan, Mexico, the Netherlands, Peru, Thailand, the Republic of Uganda, the United States, and Vietnam, respectively.
