# Supplementary material for: Genetic characteristics of human parainfluenza viruses 1–4 associated with acute lower respiratory tract infection in Chinese children, during 2015–2021
Source: Microbiol Spectr. 2024 Sep 12;12(10):e03432-23. doi: 10.1128/spectrum.03432-23 (PMC11448424; doi:10.1128/spectrum.03432-23)
Supplement: Figure S3 — Genetic distance with group and between groups of HPIV1-4. [file spectrum.03432-23-s0003.pdf]

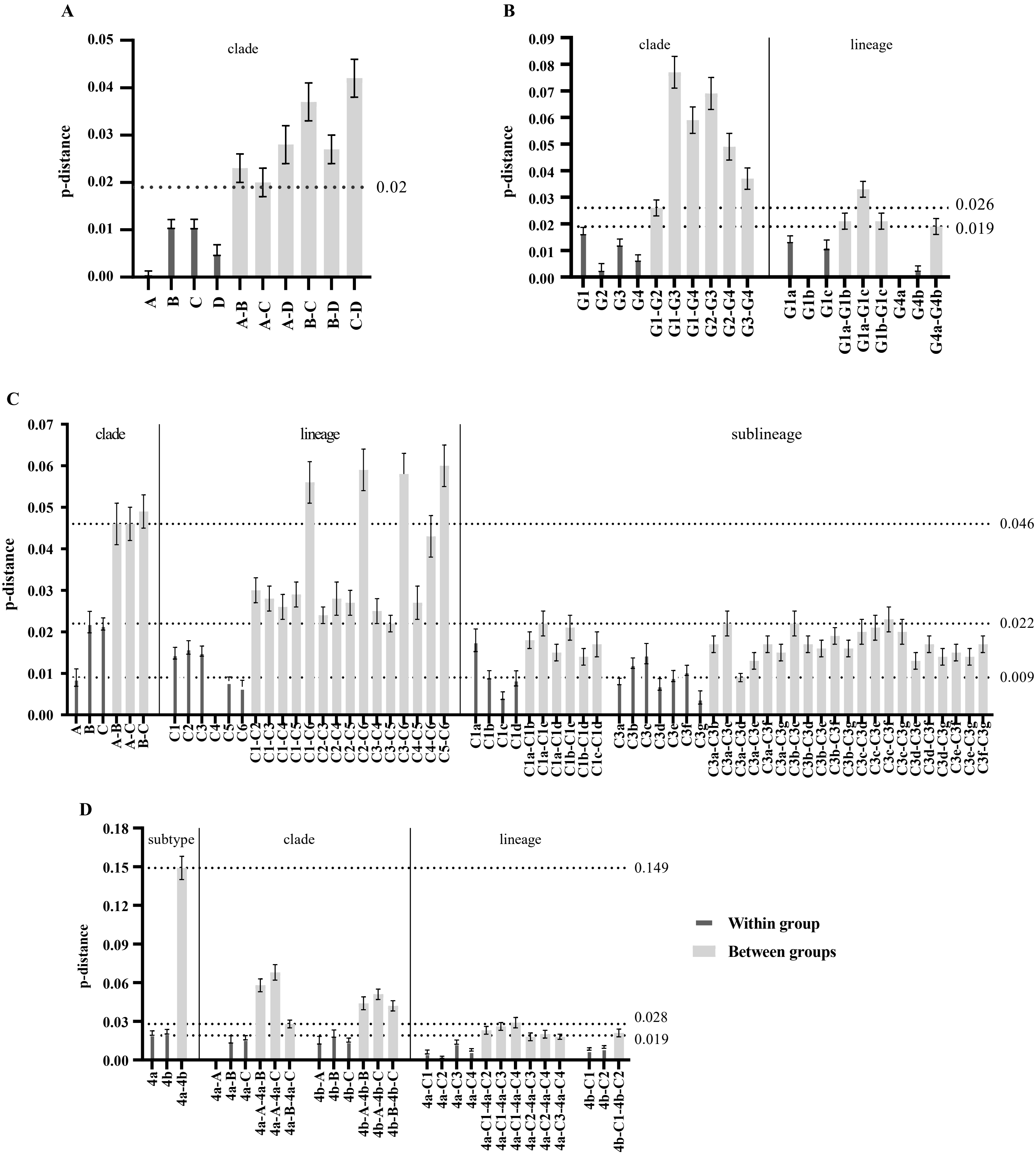

Supplementary figure 3. Genetic distance within group and between groups of HPIV1-4. Figures A-D represent genetic distance of HPIV1-4, respectively. The genetic distance (p-distance) within group and between groups was measured based on clade/lineage/sublineage assignments.
