# Supplementary material for: Genetic characteristics of human parainfluenza viruses 1–4 associated with acute lower respiratory tract infection in Chinese children, during 2015–2021
Source: Microbiol Spectr. 2024 Sep 12;12(10):e03432-23. doi: 10.1128/spectrum.03432-23 (PMC11448424; doi:10.1128/spectrum.03432-23)
Supplement: Tables S3-S5 — NSS, glycosylation site. [file spectrum.03432-23-s0004.docx]

| Supplementary Table 3. Selective pressure site in the HN protein of HPIV1-3 obtained in this study | | | | | | |  |  |  |  |  |
| --- | --- | --- | --- | --- | --- | --- | --- | --- | --- | --- | --- |
| Virus | Genotype | SLAC | |  | FUBAR | |  | FEL | |  | MEME |
|  |  | PSS | NSS |  | PSS | NSS |  | PSS | NSS |  | PSS |
| HPIV1 | B1 | N/A | 280 |  | 5 | 47, 132, 134, 162, 280, 298, 306, 349, 380, 469, 494, 506, 524, 550 |  | N/A | 162, 280, 298, 306, 349, 380, 469, 494, 506, 524, 550 |  | N/A |
|  | B2 | N/A | N/A |  | N/A | N/A |  | N/A | N/A |  | N/A |
|  | C | N/A | N/A |  | N/A | N/A |  | N/A | 138 |  | N/A |
| HPIV2 | G3 | N/A | N/A |  | N/A | 77, 204, 240, 359, 465, 498, 517 |  | N/A | 64, 77, 204, 240, 465, 498, 499, 517 |  | N/A |
|  | G1a | N/A | N/A |  | 350 | N/A |  | N/A | 93, 184, 209, 214, 221, 253, 368, 470, 525 |  | N/A |
| HPIV3 | C3a | N/A | 229, 332, 448, 513 |  | N/A | 21, 60, 92, 119, 185, 206, 223, 229, 237, 241, 268, 332, 335, 394, 407, 424, 475, 488, 491, 509, 513, 529, 571, |  | N/A | 21, 206, 223, 229, 237, 241, 332, 335, 407, 424, 488, 491, 513, 529, 571 |  | N/A |
|  | C3b | N/A | 195 |  | N/A | 93, 195, 218, 246, 379, 412, 475, 478, 539 |  | N/A | 93, 195, 218, 246, 379, 478, 539 |  | N/A |
|  | C3f | N/A | 14, 176, 470, 472, 486, 513, 526, 560 |  | N/A | 6, 14, 22, 65, 77, 82, 85, 176, 234, 239, 253, 267, 310, 319, 394, 395, 400, 444, 455, 470, 472, 486, 513, 516, 521, 526, 560, 562 |  | N/A | 6, 14, 22, 77, 82, 85, 176, 234, 253, 267, 310, 400, 444, 470, 472, 486, 513, 516, 521, 526, 560 |  | N/A |

Note: Selective pressure site inferred by at least two of algorithms was highlighted with red color.

| Supplementary Table 4 Selective pressure site in the F protein of HPIV1-3 obtained in this study | | | | | | |  |  |  |  |  |
| --- | --- | --- | --- | --- | --- | --- | --- | --- | --- | --- | --- |
| Virus | Genotype | SLAC | |  | FUBAR | |  | FEL | |  | MEME |
|  |  | PSS | NSS |  | PSS | NSS |  | PSS | NSS |  | PSS |
| HPIV1 | B1 | N/A | 88, 228 |  | N/A | 9, 15, 16, 24, 41, 51, 77, 85, 88, 116, 178, 204, 228, 377, 515, 525, 531, 555 |  | N/A | 24, 41, 51, 77, 88, 116, 228, 377, 531, 555 |  | N/A |
|  | B2 | N/A | N/A |  | N/A | N/A |  | N/A | 124 |  | N/A |
|  | C | N/A | N/A |  | N/A | N/A |  | N/A | 103, 506 |  | N/A |
| HPIV2 | G3 | N/A | N/A |  | N/A | 22, 163, 279, 321, 512 |  | N/A | 22, 100, 163, 279, 321, 512 |  | N/A |
|  | G1a | N/A | N/A |  | 513 | 300 |  | N/A | 26, 39, 300, 386, 449 |  | N/A |
| HPIV3 | C3a | N/A | 59, 124, 135, 383, 395, 398 |  | N/A | 9, 35, 59, 60, 65, 86, 90, 99,117, 124, 135, 155, 162, 186, 200, 260, 277, 293, 318, 361, 383, 395, 398, 417, 421, 441, 450, 457, 504, 507, 527 |  | N/A | 9, 35, 55, 59, 99, 117, 124, 135, 171, 186, 194, 200, 260, 277, 318, 361, 383, 395, 398, 417, 421, 450, 457, 507, 527 |  | 3 |
|  | C3b | N/A | N/A |  | N/A | 162, 180, 286, 323, 395, 433, 480, 531 |  | N/A | 286, 395, 433, 531 |  | N/A |
|  | C3f | N/A | 95, 128, 318, 392, 395, 396, 398, 412, 453, 490 |  | N/A | 13, 46, 51, 95, 99, 109, 117, 128, 139, 152, 155, 164, 183, 188, 194, 198, 199, 209, 262, 318, 332, 335, 336, 374, 385, 386, 392, 394, 395, 396, 397, 398, 412, 414, 417, 453, 482, 490, 525 |  | N/A | 46, 95, 99, 109, 117, 126, 128, 139, 155, 164, 185, 188, 194, 199, 209, 262, 318, 332, 335, 336, 344, 374, 385, 386, 392, 394, 395, 396, 398, 412, 414, 417, 453, 490, 525 |  | N/A |

Note: Selective pressure site inferred by at least two of algorithms was highlighted with red color.

Supplementary Table 5. N-glycosylation and O-glycosylation site in the HN and F proteins of HPIV1-4 identified in this study

| NO. | Strains | HN | | |  | | F | |
| --- | --- | --- | --- | --- | --- | --- | --- | --- |
|  |  | N-glycosylation site | | O-glycosylation site | |  | N-glycosylation site | O-glycosylation site |
| 1 | HPIV1/China_Beijing/BCH20150558/2015 | 19 NST, 173 NIS, 277 NET, 361 NQS, 499 NTS, 504 NPT | | 151 |  | | 241 NIT | 102, 103, 453 |
| 2 | HPIV1/China_Beijing/BCH20160432/2016 | 19 NST, 173 NIS, 277 NET, 361 NQS, 499 NTS, 504 NPT | | 147, 151 |  | | 241 NIT | 102, 103, 453 |
| 3 | HPIV1/China Beijing/BCH20160495/2016 | 19 NST, 173 NIS, 277 NET, 361 NQS, 499 NTS, 504 NPT, 511 NTS | | 74, 79, 151 |  | | 241 NIT, 529 NST | 102, 103, 453 |
| 4 | HPIV1/China_Beijing/BCH20170219/2017 | 19 NST, 173 NIS, 277 NET, 361 NQS, 499 NTS, 504 NPT | | 151 |  | | 241 NIT | 102, 103, 453 |
| 5 | HPIV1/China_Beijing/BCH20170252/2017 | 19 NST, 173 NIS, 277 NET, 361 NQS, 499 NTS, 504 NPT | | 79, 151 |  | | 241 NIT | 102 |
| 6 | HPIV1/China_Beijing/BCH20170345/2017 | 19 NST, 173 NIS, 277 NET, 361 NQS, 499 NTS, 504 NPT | | 151 |  | | 241 NIT | 102, 103, 453 |
| 7 | HPIV1/China Beijing/BCH20170406/2017 | 19 NST, 173 NIS, 277 NET, 361 NQS, 499 NTS, 504 NPT | | 151 |  | | 241 NIT | 102, 103, 453 |
| 8 | HPIV1/China_Beijing/BCH20170584/2017 | 19 NST, 173 NIS, 277 NET, 361 NQS, 499 NTS, 504 NPT | | 151 |  | | 241 NIT | 102, 103, 453 |
| 9 | HPIV1/China_Beijing/BCH20180405/2018 | 19 NST, 173 NIS, 277 NET, 361 NQS, 499 NTS, 504 NPT | | 151 |  | | 241 NIT | 102, 103, 453 |
| 10 | HPIV1/China_Beijing/BCH20180721/2018 | 19 NST, 173 NIS, 277 NET, 361 NQS, 499 NTS, 504 NPT | | N/A |  | | 241 NIT | 102, 103, 453 |
| 11 | HPIV1/China_Beijing/BCH20180770/2018 | 19 NST, 173 NIS, 277 NET, 361 NQS, 499 NTS, 504 NPT | | 151 |  | | 241 NIT | 102, 103, 453 |
| 12 | HPIV1/China_Beijing/BCH20180797/2018 | 19 NST, 173 NIS, 277 NET, 361 NQS, 499 NTS, 504 NPT | | 151 |  | | 241 NIT | 102, 103, 453 |
| 13 | HPIV1/China_Beijing/BCH20180919/2018 | 19 NST, 173 NIS, 277 NET, 361 NQS, 499 NTS, 504 NPT | | 151 |  | | 241 NIT | 102, 103, 453 |
| 14 | HPIV1/China_Beijing/BCH19055/2019 | 19 NST, 173 NIS, 277 NET, 361 NQS, 499 NTS, 504 NPT | | 79, 151 |  | | 241 NIT | 102, 103, 453 |
| 15 | HPIV1/China_Beijing/BCH19192/2019 | 19 NST, 173 NIS, 277 NET, 361 NQS, 499 NTS, 504 NPT | | 151 |  | | 241 NIT | 102, 103, 453 |
| 16 | HPIV1/China_Beijing/BCH19213/2019 | 19 NST, 173 NIS, 277 NET, 361 NQS, 499 NTS, 504 NPT | | 79, 151 |  | | 241 NIT | 102, 103, 453 |
| 17 | HPIV1/China_Beijing/BCH20200226/2020 | 19 NST, 173 NIS, 277 NET, 361 NQS, 499 NTS, 504 NPT | | 79, 151 |  | | 241 NIT | 102, 103, 453 |
| 18 | HPIV1/China_Beijing/BCH20200698/2020 | 19 NST, 173 NIS, 277 NET, 361 NQS, 499 NTS, 504 NPT | | 151 |  | | 241 NIT | 102, 103, 453 |
| 19 | HPIV1/China_Beijing/BCH20200781/2020 | 19 NST, 173 NIS, 277 NET, 361 NQS, 499 NTS, 504 NPT | | 151 |  | | 241 NIT | 102, 103, 453 |
| 20 | HPIV1/China_Beijing/BCH20200782/2020 | 19 NST, 173 NIS, 277 NET, 361 NQS, 499 NTS, 504 NPT | | 151 |  | | 241 NIT | 102, 103, 453 |
| 21 | HPIV1/China_Beijing/BCH20200796/2021 | 19 NST, 173 NIS, 277 NET, 361 NQS, 499 NTS, 504 NPT | | 79, 151 |  | | 241 NIT | 102 |
| 22 | HPIV1/China_Beijing/BCH20210017/2021 | 19 NST, 173 NIS, 277 NET, 361 NQS, 499 NTS, 504 NPT | | 79, 151 |  | | 241 NIT | 102, 103, 453 |
| 23 | HPIV1/China_Beijing/BCH20210081/2021 | 19 NST, 173 NIS, 277 NET, 361 NQS, 499 NTS, 504 NPT | | 151 |  | | 241 NIT | 102, 103, 453 |
| 24 | HPIV1/China_Beijing/BCH20210093/2021 | 19 NST, 173 NIS, 277 NET, 361 NQS, 499 NTS, 504 NPT | | 151 |  | | 241 NIT | 102, 103 |
| 25 | HPIV1/China_Beijing/BCH20210111/2021 | 19 NST, 173 NIS, 277 NET, 361 NQS, 499 NTS, 504 NPT | | 151 |  | | 241 NIT | 102, 103 |
| 26 | HPIV1/China_Beijing/BCH20210137/2021 | 19 NST, 77 NKS, 173 NIS, 277 NET, 361 NQS, 499 NTS, 504 NPT | | 151 |  | | 241 NIT | 102, 103 |
| 27 | HPIV1/China Beijing/BCH288/2021 | 19 NST, 173 NIS, 277 NET, 361 NQS, 499 NTS, 504 NPT | | 79, 151 |  | | 241 NIT | 102, 103 |
| 28 | HPIV1/China_Guizhou/GY18189/2018 | 19 NST, 173 NIS, 277 NET, 361 NQS, 499 NTS, 504 NPT | | 79, 151 |  | | 241 NIT | 102, 103, 453 |
| 29 | HPIV1/China_Guizhou/GY18190/2018 | 19 NST, 173 NIS, 277 NET, 361 NQS, 499 NTS, 504 NPT | | 151 |  | | 241 NIT | 102, 103, 453 |
| 30 | HPIV1/China_Ningxia/YC17073/2017 | 19 NST, 173 NIS, 277 NET, 361 NQS, 499 NTS, 504 NPT | | 79, 151 |  | | 241 NIT | 102,103 |
| 31 | HPIV1/China_Ningxia/YC17088/2017 | 19 NST, 173 NIS, 277 NET, 361 NQS, 499 NTS, 504 NPT | | 79, 151 |  | | 100 NDT,241 NIT | 102, 103, 453 |
| 32 | HPIV1/China_Ningxia/YC17091/2017 | 19 NST,173 NIS, 277 NET, 361 NQS, 499 NTS, 504 NPT, 511 NTS | | 74, 79, 151 |  | | 241 NIT, 529 NST | 102, 103 |
| 33 | HPIV1/China_Ningxia/YC18090/2018 | 19 NST,173 NIS, 277 NET, 361 NQS, 499 NTS, 504 NPT, 511 NTS | | 151 |  | | 241 NIT, 529 NST | 102, 103 |
| 34 | HPIV1/China_Ningxia/YC18091/2018 | 19 NST,173 NIS, 277 NET, 361 NQS, 499 NTS, 504 NPT, 511 NTS | | 151 |  | | 241 NIT, 529 NST | 102, 103 |
| 35 | HPIV1/China_Ningxia/YC18101/2018 | 19 NST, 173 NIS, 277 NET, 361 NQS, 499 NTS, 504 NPT | | 151 |  | | 241 NIT | 102, 103, 453 |
| 36 | HPIV1/China_Ningxia/YC18154/2018 | 19 NST, 173 NIS, 277 NET, 361 NQS, 499 NTS, 504 NPT | | 151 |  | | 241 NIT | 102, 103, 453 |
| 37 | HPIV1/China_Ningxia/YC19062/2019 | 19 NST, 173 NIS, 277 NET, 361 NQS, 499 NTS, 504 NPT | | 74, 79, 151 |  | | 241 NIT | 102, 103 |
| 38 | HPIV1/China_Ningxia/YC19076/2019 | 19 NST, 173 NIS, 277 NET, 361 NQS, 499 NTS, 504 NPT | | 74, 79, 151 |  | | 241 NIT | 102 |
| 39 | HPIV1/China_Ningxia/YC19111/2019 | 19 NST, 173 NIS, 277 NET, 361 NQS, 499 NTS, 504 NPT | | 151 |  | | 241 NIT | 102, 103, 453 |
| 40 | HPIV1/China_Ningxia/YC19192/2020 | 19 NST, 173 NIS, 277 NET, 361 NQS, 499 NTS, 504 NPT | | 151 |  | | 241 NIT | 102, 103, 453 |
| 41 | HPIV1/China_Zhejiang/WZ17106/2018 | 19 NST, 173 NIS, 277 NET, 361 NQS, 499 NTS, 504 NPT | | 74, 79, 151 |  | | 241 NIT | 102, 103, 453 |
| 42 | HPIV1/China_Zhejiang/WZ17141/2018 | 19 NST, 173 NIS, 277 NET, 361 NQS, 499 NTS, 504 NPT | | 151 |  | | 241 NIT | 102, 103, 453 |
| 43 | HPIV1/China_Zhejiang/WZ17271/2018 | 19 NST, 173 NIS, 277 NET, 361 NQS, 499 NTS, 504 NPT | | 151 |  | | 241 NIT | 102, 103, 453 |
| 44 | HPIV1/China_Zhejiang/WZ17307/2018 | 19 NST, 173 NIS, 277 NET, 361 NQS, 499 NTS, 504 NPT | | 74, 79, 151 |  | | 241 NIT | 102, 103, 453 |
| 45 | HPIV1/China_Zhejiang/WZ17313/2018 | 19 NST, 173 NIS, 277 NET, 361 NQS, 499 NTS, 504 NPT | | 79, 151 |  | | 241 NIT | 102, 103, 453 |
| 46 | HPIV2/China Beijing/BCH20160149/2016 | 6 NLS, 272 NDT, 284 NTT, 316 NGT, 335 NIT, 341 NSS, 454 NAT, 501 NRT, 517 NTT, | 325, 326, 343, 351,442 | |  | | 65 NGT, 69 NIT, 77 NVT, 90 NLS, 431 NAT | 156 |
| 47 | HPIV2/China Beijing/BCH20170155/2017 | 6 NLS, 115 NCT, 272 NDT, 284 NTT, 335 NIT, 341 NSS, 454 NAT, 501 NRT, 517 NTT, | 325, 326, 332, 343, 442 | |  | | 65 NGT, 69 NIT, 77 NVT, 90 NLS, 431 NAT | None |
| 48 | HPIV2/China_Beijing/BCH20170249/2017 | 6 NLS, 272 NDT, 284 NTT, 316 NGT, 335 NIT, 341 NSS, 454 NAT, 501 NRT, 517 NTT, | 325, 326, 343, 344, 351, 442 | |  | | 65 NGT, 69 NIT, 77 NVT, 90 NLS, 431 NAT | 156 |
| 49 | HPIV2/China_Beijing/BCH20170272/2017 | 6 NLS, 272 NDT, 284 NTT, 335 NIT, 341 NSS, 454 NAT, 501 NRT, 517 NTT, | 325, 326, 332, 343, 442 | |  | | 65 NGT, 69 NIT, 77 NVT, 90 NLS, 431 NAT | None |
| 50 | HPIV2/China Beijing/BCH20170307/2017 | 6 NLS, 272 NDT, 284 NTT, 316 NGT, 335 NIT, 341 NSS, 454 NAT, 501 NRT, 517 NTT, | 325, 326, 332, 343, 348, 442 | |  | | 65 NGT, 69 NIT, 77 NVT, 90 NLS, 431 NAT | None |
| 51 | HPIV2/China Beijing/BCH20170457/2017 | 6 NLS, 272 NDT, 284 NTT, 316 NGT, 335 NIT, 341 NSS, 454 NAT, 501 NRT, 517 NTT, | 325, 326, 343, 344, 351, 442 | |  | | 65 NGT, 69 NIT, 77 NVT, 90 NLS, 431 NAT | 156 |
| 52 | HPIV2/China Beijing/BCH19212/2019 | 6 NLS, 272 NDT, 284 NTT, 316 NGT, 335 NIT, 341 NSS, 454 NAT, 501 NRT, 517 NTT, | 325, 326, 343, 344, 351, 442 | |  | | 65 NGT, 69 NIT, 77 NVT, 90 NLS, 431 NAT | 156 |
| 53 | HPIV2/China Ningxia/YC17068/2017 | 6 NLS, 272 NDT, 284 NTT, 316 NGT, 335 NIT, 341 NSS, 454 NAT, 501 NRT, 517 NTT, | 325, 326, 343, 351, 442 | |  | | 65 NGT, 69 NIT, 77 NVT, 90 NLS, 431 NAT | 156 |
| 54 | HPIV2/China Zhejiang/WZ17441/2019 | 6 NLS, 272 NDT, 284 NTT, 316 NGT, 335 NIT, 341 NSS, 454 NAT, 501 NRT, 517 NTT, | 325, 326, 442 | |  | | 65 NGT, 69 NIT, 77 NVT, 90 NLS, 431 NAT | 156 |
| 55 | HPIV3/China_Beijing/BCH20150780/2015 | 308 NIS, 485 NPT, 523 NKT | 126, 143, 352, 353, 359 | |  | | 238 NIT, 359 NIS, 446 NNS | 245, 246 |
| 56 | HPIV3/China_Beijing/BCH20150797/2015 | 308 NIS, 485 NPT, 523 NKT | 126, 143, 352, 353, 359 | |  | | 238 NIT, 359 NIS, 446 NNS, 508 NVT | 245, 246 |
| 57 | HPIV3/China_Beijing/BCH20150924/2015 | 308 NIS, 485 NPT, 523 NKT | 126, 143, 352, 353, 359 | |  | | 238 NIT, 359 NIS, 446 NNS, 508 NVT | 245, 246 |
| 58 | HPIV3/China_Beijing/BCH20151012/2015 | 308 NIS, 485 NPT, 523 NKT | 126, 143, 352, 353, 359 | |  | | 238 NIT, 359 NIS, 446 NNS, 508 NVT | 245, 246 |
| 59 | HPIV3/China_Beijing/BCH20151067/2015 | 308 NIS, 485 NPT, 523 NKT | 126, 143, 352, 353, 359 | |  | | 238 NIT, 359 NIS, 446 NNS, 508 NVT | 245, 246 |
| 60 | HPIV3/China_Beijing/BCH20160015/2016 | 308 NIS, 485 NPT, 523 NKT | 126, 143, 352, 353, 359 | |  | | 238 NIT, 359 NIS, 446 NNS, 508 NVT | 245, 246 |
| 61 | HPIV3/China_Beijing/BCH20160110/2016 | 308 NIS, 485 NPT, 523 NKT | 126, 143, 352, 353, 359 | |  | | 238 NIT, 359 NIS, 508 NIT | 245, 246 |
| 62 | HPIV3/China_Beijing/BCH20160184/2016 | 308 NIS, 485 NPT, 523 NKT | 126, 143, 352, 353, 359 | |  | | 238 NIT, 359 NIS, 446 NNS, 508 NVT | 245, 246 |
| 63 | HPIV3/China_Beijing/BCH20160189/2016 | 308 NIS, 485 NPT, 523 NKT | 126, 143, 352, 353, 359 | |  | | 238 NIT, 359 NIS, 446 NNS, 508 NVT | 245, 246 |
| 64 | HPIV3/China_Beijing/BCH20160445/2016 | 308 NIS, 485 NPT, 523 NKT | 126, 143, 352, 353, 359 | |  | | 238 NIT, 359 NIS, 446 NNS, 508 NVT | 245, 246 |
| 65 | HPIV3/China_Beijing/BCH20160620/2016 | 308 NIS, 485 NPT, 523 NKT | 126, 143, 352, 353, 359 | |  | | 238 NIT, 359 NIS, 446 NNS, 508 NVT | 245, 246 |
| 66 | HPIV3/China Beijing/BCH17080/2017 | 308 NIS, 485 NPT, 523 NKT | 126, 143, 352, 353, 359 | |  | | 238 NIT, 359 NIS, 446 NNS, 508 NVT | 245, 246 |
| 67 | HPIV3/China_Beijing/BCH20170125/2017 | 308 NIS, 485 NPT, 523 NKT | 126, 143, 352, 353, 359 | |  | | 238 NIT, 359 NIS, 446 NNS, 508 NVT | 245, 246 |
| 68 | HPIV3/China_Beijing/BCH20170290/2017 | 308 NIS, 485 NPT, 523 NKT | 126, 143, 352, 353, 359 | |  | | 238 NIT, 359 NIS, 446 NNS, 508 NVT | 245, 246 |
| 69 | HPIV3/China_Beijing/BCH20170298/2017 | 308 NIS, 485 NPT, 523 NKT | 126, 143, 352, 353, 359 | |  | | 238 NIT, 359 NIS, 446 NNS, 508 NVT | 245, 246 |
| 70 | HPIV3/China_Beijing/BCH20170368/2017 | 308 NIS, 485 NPT, 523 NKT | 126, 143, 352, 353, 359 | |  | | 238 NIT, 359 NIS, 508 NIT | 245, 246 |
| 71 | HPIV3/China_Beijing/BCH20170451/2017 | 308 NIS, 485 NPT, 523 NKT | 126, 143, 352, 353, 359 | |  | | 238 NIT, 359 NIS, 446 NNS, 508 NVT | 245, 246 |
| 72 | HPIV3/China_Beijing/BCH20170582/2017 | 308 NIS, 485 NPT, 523 NKT | 126, 143, 352, 353, 359 | |  | | 238 NIT, 359 NIS, 446 NNS, 508 NVT | 245, 246 |
| 73 | HPIV3/China Beijing/BCH18024/2018 | 308 NIS, 485 NPT, 523 NKT | 126, 143, 352, 353, 359 | |  | | 238 NIT, 359 NIS, 446 NNS, 508 NVT | 245, 246 |
| 74 | HPIV3/China_Beijing/BCH20190096/2019 | 308 NIS, 485 NPT, 523 NKT | 126, 143, 352, 353, 359 | |  | | 238 NIT, 359 NIS, 446 NNS, 508 NVT | 245, 246 |
| 75 | HPIV3/China_Beijing/BCH20190234/2019 | 308 NIS, 485 NPT, 523 NKT | 126, 143, 352, 353, 359 | |  | | 238 NIT, 359 NIS, 446 NNS, 508 NVT | 245, 246 |
| 76 | HPIV3/China_Beijing/BCH20190337/2019 | 308 NIS, 485 NPT, 523 NKT | 126, 143, 352, 353, 359 | |  | | 238 NIT, 359 NIS, 446 NNS, 508 NVT | 245, 246 |
| 77 | HPIV3/China_Beijing/BCH20190362/2019 | 308 NIS, 485 NPT, 523 NKT | 126, 143, 352 | |  | | 238 NIT, 359 NIS, 446 NNS, 508 NVT | 245, 246 |
| 78 | HPIV3/China_Beijing/BCH20190390/2019 | 308 NIS, 485 NPT, 523 NKT | 126, 143, 352, 353, 359 | |  | | 238 NIT, 359 NIS, 446 NNS, 508 NVT | 245, 246 |
| 79 | HPIV3/China_Beijing/BCH20190502/2019 | 308 NIS, 485 NPT, 523 NKT | 126, 143, 352, 353, 359 | |  | | 238 NIT, 359 NIS, 446 NNS, 508 NVT | 245, 246 |
| 80 | HPIV3/China_Beijing/BCH20190558/2019 | 308 NIS, 485 NPT, 523 NKT | 126, 143, 161, 165, 352, 353, 359 | |  | | 238 NIT, 359 NIS, 446 NNS, 508 NVT | 245, 246 |
| 81 | HPIV3/China_Beijing/BCH20190563/2019 | 308 NIS, 485 NPT, 523 NKT | 126, 143, 352, 353, 359 | |  | | 238 NIT, 359 NIS, 446 NNS, 508 NVT | 245, 246 |
| 82 | HPIV3/China_Beijing/BCH20190565/2019 | 308 NIS, 485 NPT, 523 NKT | 126, 143, 352, 353, 359 | |  | | 238 NIT, 359 NIS, 446 NNS, 508 NVT | 245, 246 |
| 83 | HPIV3/China_Beijing/BCH20190570/2019 | 308 NIS, 485 NPT, 523 NKT | 126, 143, 352, 353, 359 | |  | | 238 NIT, 359 NIS, 446 NNS, 508 NVT | 245, 246 |
| 84 | HPIV3/China_Beijing/BCH20190595/2019 | 308 NIS, 485 NPT, 523 NKT | 126, 143, 352, 353, 359 | |  | | 238 NIT, 359 NIS, 446 NNS, 508 NVT | 245, 246 |
| 85 | HPIV3/China_Beijing/BCH20190607/2019 | 308 NIS, 485 NPT, 523 NKT | 126, 143, 352, 353, 359 | |  | | 238 NIT, 359 NIS, 446 NNS, 508 NVT | 245, 246 |
| 86 | HPIV3/China_Beijing/BCH20190663/2019 | 308 NIS, 485 NPT, 523 NKT | 126, 143, 352, 353, 359 | |  | | 238 NIT, 359 NIS, 446 NNS, 508 NVT | 245, 246 |
| 87 | HPIV3/China_Beijing/BCH20190727/2019 | 308 NIS, 485 NPT, 523 NKT | 126, 143, 352, 353, 359 | |  | | 238 NIT, 359 NIS, 446 NNS, 508 NVT | 245, 246 |
| 88 | HPIV3/China_Beijing/BCH20190728/2019 | 308 NIS, 485 NPT, 523 NKT | 126, 143, 352, 353, 359 | |  | | 238 NIT, 359 NIS, 446 NNS, 508 NVT | 245, 246 |
| 89 | HPIV3/China_Beijing/BCH20190730/2019 | 308 NIS, 485 NPT, 523 NKT | 126, 143, 352, 353, 359 | |  | | 238 NIT, 359 NIS, 446 NNS, 508 NVT | 245, 246 |
| 90 | HPIV3/China_Beijing/BCH20190733/2019 | 308 NIS, 485 NPT, 523 NKT | 126, 143, 352, 353, 359 | |  | | 238 NIT, 359 NIS, 446 NNS, 508 NVT | 245, 246 |
| 91 | HPIV3/China_Beijing/BCH20190734/2019 | 308 NIS, 485 NPT, 523 NKT | 126, 143, 352, 353, 359 | |  | | 238 NIT, 359 NIS, 446 NNS, 508 NVT | 245, 246 |
| 92 | HPIV3/China_Beijing/BCH20190743/2019 | 308 NIS, 485 NPT, 523 NKT | 126, 143, 352, 353, 359 | |  | | 238 NIT, 359 NIS, 446 NNS, 508 NVT | 245, 246 |
| 93 | HPIV3/China_Beijing/BCH20190746/2019 | 308 NIS, 485 NPT, 523 NKT | 126, 143, 352, 353, 359 | |  | | 238 NIT, 359 NIS, 446 NNS, 508 NVT | 245, 246 |
| 94 | HPIV3/China_Beijing/BCH20190753/2019 | 308 NIS, 485 NPT, 523 NKT | 126, 143, 352, 353, 359 | |  | | 238 NIT, 359 NIS, 446 NNS, 508 NVT | 245, 246 |
| 95 | HPIV3/China_Beijing/BCH20190758/2019 | 308 NIS, 485 NPT, 523 NKT | 126, 143, 352, 353, 359 | |  | | 238 NIT, 359 NIS, 446 NNS, 508 NVT | 245, 246 |
| 96 | HPIV3/China_Beijing/BCH20190766/2019 | 308 NIS, 485 NPT, 523 NKT | 126, 143, 352, 353, 359 | |  | | 238 NIT, 359 NIS, 446 NNS, 508 NVT | 245, 246 |
| 97 | HPIV3/China_Beijing/BCH20190767/2019 | 308 NIS, 485 NPT, 523 NKT | 126, 143, 352, 353, 359 | |  | | 238 NIT, 359 NIS, 446 NNS, 508 NVT | 245, 246 |
| 98 | HPIV3/China_Beijing/BCH20190768/2019 | 308 NIS, 485 NPT, 523 NKT | 126, 143, 352, 353, 359 | |  | | 238 NIT, 359 NIS, 446 NNS, 508 NVT | 245, 246 |
| 99 | HPIV3/China_Beijing/BCH20190829/2019 | 308 NIS, 485 NPT, 523 NKT | 126, 143, 352, 353, 359 | |  | | 238 NIT, 359 NIS, 446 NNS, 508 NVT | 245, 246 |
| 100 | HPIV3/China_Beijing/BCH20190839/2019 | 308 NIS, 485 NPT, 523 NKT | 126, 143, 352, 353, 359 | |  | | 238 NIT, 359 NIS, 446 NNS, 508 NVT | 245, 246 |
| 101 | HPIV3/China_Beijing/BCH20190861/2019 | 308 NIS, 485 NPT, 523 NKT | 126, 143, 352, 353, 359 | |  | | 238 NIT, 359 NIS, 446 NNS, 508 NVT | 245, 246 |
| 102 | HPIV3/China_Beijing/BCH20190878/2019 | 308 NIS, 485 NPT, 523 NKT | 126, 143, 352, 353, 359 | |  | | 238 NIT, 359 NIS, 446 NNS, 508 NVT | 245, 246 |
| 103 | HPIV3/China_Beijing/BCH20190903/2019 | 308 NIS, 485 NPT, 523 NKT | 126, 143, 352, 353, 359 | |  | | 238 NIT, 359 NIS, 446 NNS, 508 NVT | 245, 246 |
| 104 | HPIV3/China_Beijing/BCH20190912/2019 | 308 NIS, 485 NPT, 523 NKT | 126, 143, 352, 353, 359 | |  | | 238 NIT, 359 NIS, 446 NNS, 508 NVT | 245, 246 |
| 105 | HPIV3/China_Beijing/BCH20190973/2019 | 308 NIS, 485 NPT, 523 NKT | 126, 143, 352, 353, 359 | |  | | 238 NIT, 359 NIS, 446 NNS, 508 NVT | 245, 246 |
| 106 | HPIV3/China_Beijing/BCH20200037/2020 | 308 NIS, 485 NPT, 523 NKT | 126, 143, 352, 353, 359 | |  | | 238 NIT, 359 NIS, 446 NNS, 508 NVT | 245, 246 |
| 107 | HPIV3/China_Beijing/BCH20200041/2020 | 308 NIS, 485 NPT, 523 NKT | 126, 143, 352, 353, 359 | |  | | 238 NIT, 359 NIS, 446 NNS, 508 NVT | 245, 246 |
| 108 | HPIV3/China_Beijing/BCH20200077/2020 | 308 NIS, 485 NPT, 523 NKT | 126, 143, 352, 353, 359 | |  | | 238 NIT, 359 NIS, 446 NNS, 508 NVT | 245, 246 |
| 109 | HPIV3/China_Beijing/BCH20200090/2020 | 308 NIS, 485 NPT, 523 NKT | 126, 143, 352, 353, 359 | |  | | 238 NIT, 359 NIS, 446 NNS, 508 NVT | 99, 245, 246 |
| 110 | HPIV3/China_Beijing/BCH20200440/2020 | 308 NIS, 485 NPT, 523 NKT | 126, 143, 352, 353, 359 | |  | | 238 NIT, 359 NIS, 446 NNS, 508 NVT | 245, 246 |
| 111 | HPIV3/China_Beijing/BCH20200450/2020 | 308 NIS, 485 NPT, 523 NKT | 126, 143, 352, 353, 359 | |  | | 238 NIT, 359 NIS, 446 NNS, 508 NVT | 245, 246 |
| 112 | HPIV3/China_Guangdong/GZFE17033/2018 | 308 NIS, 485 NPT, 523 NKT | 126, 143, 352, 353, 359 | |  | | 238 NIT, 359 NIS, 446 NNS, 508 NVT | 245, 246 |
| 113 | HPIV3/China_Guangdong/GZFE17038/2018 | 308 NIS, 485 NPT, 523 NKT | 126, 143, 352, 353, 359 | |  | | 238 NIT, 359 NIS, 446 NNS, 508 NVT | 245, 246 |
| 114 | HPIV3/China_Guangdong/GZFE17044/2018 | 308 NIS, 485 NPT, 523 NKT | 126, 143, 352, 353, 359 | |  | | 238 NIT, 359 NIS, 446 NNS, 508 NVT | 245, 246 |
| 115 | HPIV3/China_Guangdong/GZFE17046/2018 | 308 NIS, 485 NPT, 523 NKT | 126, 143, 352, 353, 359 | |  | | 238 NIT, 359 NIS, 446 NNS, 508 NVT | 245, 246 |
| 116 | HPIV3/China_Guangdong/GZFE17049/2018 | 308 NIS, 485 NPT, 523 NKT | 126, 143, 352, 353, 359 | |  | | 238 NIT, 359 NIS, 446 NNS, 508 NVT | 245, 246 |
| 117 | HPIV3/China_Guangdong/GZFE17067/2018 | 308 NIS, 485 NPT, 523 NKT | 126, 143, 352, 353, 359 | |  | | 238 NIT, 359 NIS, 446 NNS, 508 NVT | 245, 246 |
| 118 | HPIV3/China_Guangdong/GZFE17096/2018 | 308 NIS, 485 NPT, 523 NKT | 126, 143, 352, 353, 359 | |  | | 238 NIT, 359 NIS, 446 NNS, 508 NVT | 245, 246 |
| 119 | HPIV3/China_Guangdong/GZFE17127/2018 | 308 NIS, 485 NPT, 523 NKT | 126, 143, 352, 353, 359 | |  | | 238 NIT, 359 NIS, 446 NNS, 508 NVT | 245, 246 |
| 120 | HPIV3/China_Guangdong/GZFE17159/2018 | 308 NIS, 485 NPT, 523 NKT | 126, 143, 352, 353, 359 | |  | | 238 NIT, 359 NIS, 446 NNS, 508 NVT | 245, 246 |
| 121 | HPIV3/China_Guangdong/GZFE18027/2018 | 308 NIS, 485 NPT, 523 NKT | 126, 143, 352, 353, 359 | |  | | 238 NIT, 359 NIS, 446 NNS, 508 NVT | 245, 246 |
| 122 | HPIV3/China_Guangdong/GZFE18089/2018 | 308 NIS, 485 NPT, 523 NKT | 126, 143, 352, 353, 359 | |  | | 238 NIT, 359 NIS, 446 NNS, 508 NVT | 245, 246 |
| 123 | HPIV3/China_Guangdong/GZFE19008/2019 | 308 NIS, 485 NPT, 523 NKT | 126, 143, 352, 353, 359 | |  | | 238 NIT, 359 NIS, 446 NNS, 508 NVT | 245, 246 |
| 124 | HPIV3/China_Guangdong/GZFE19065/2019 | 308 NIS, 485 NPT, 523 NKT | 126, 143, 352, 353, 359 | |  | | 238 NIT, 359 NIS, 446 NNS, 508 NVT | 245, 246 |
| 125 | HPIV3/China_Guangdong/GZFE19196/2019 | 308 NIS, 485 NPT, 523 NKT | 126, 143, 352, 353, 359 | |  | | 238 NIT, 359 NIS, 446 NNS, 508 NVT | 245, 246 |
| 126 | HPIV3/China_Guiyang/GY17020/2017 | 308 NIS, 485 NPT, 523 NKT | 126, 143, 352, 353, 359 | |  | | 238 NIT, 359 NIS, 446 NNS, 508 NVT | 245, 246 |
| 127 | HPIV3/China_Guiyang/GY17027/2017 | 308 NIS, 485 NPT, 523 NKT | 126, 143, 352, 353, 359 | |  | | 238 NIT, 359 NIS, 446 NNS, 508 NVT | 245, 246 |
| 128 | HPIV3/China_Guiyang/GY18077/2018 | 308 NIS, 485 NPT, 523 NKT | 126, 143, 352, 353, 359 | |  | | 238 NIT, 359 NIS, 446 NNS, 508 NVT | 245, 246 |
| 129 | HPIV3/China_Guiyang/GY18090/2018 | 308 NIS, 485 NPT, 523 NKT | 126, 143, 352, 353, 359 | |  | | 238 NIT, 359 NIS, 446 NNS, 508 NVT | 245, 246 |
| 130 | HPIV3/China_Guiyang/GY19052/2019 | 308 NIS, 485 NPT, 523 NKT | 126, 143, 352, 353, 359 | |  | | 238 NIT, 359 NIS, 446 NNS, 508 NVT | 245, 246 |
| 131 | HPIV3/China_Liaoning/SJ18114/2018 | 308 NIS, 485 NPT, 523 NKT | 126, 143, 352, 353, 359 | |  | | 238 NIT, 359 NIS, 446 NNS, 508 NVT | 245, 246 |
| 132 | HPIV3/China_Ningxia/YC17071/2017 | 308 NIS, 485 NPT, 523 NKT | 126, 143, 352, 353, 359 | |  | | 238 NIT, 359 NIS, 446 NNS, 508 NVT | 245, 246 |
| 133 | HPIV3/China_Ningxia/YC17084/2017 | 308 NIS, 485 NPT, 523 NKT | 126, 143, 352, 353, 359 | |  | | 238 NIT, 359 NIS, 446 NNS, 508 NVT | 245, 246 |
| 134 | HPIV3/China_Ningxia/YC17193/2017 | 308 NIS, 485 NPT, 523 NKT | 126, 143, 352, 353, 359 | |  | | 238 NIT, 359 NIS, 446 NNS, 508 NVT | 245, 246 |
| 135 | HPIV3/China_Ningxia/YC18058/2018 | 30 NKT, 308 NIS, 485 NPT, 523 NKT | 126, 143, 352, 353, 359 | |  | | 238 NIT, 359 NIS, 446 NNS, 508 NVT | 245, 246 |
| 136 | HPIV3/China_Ningxia/YC18065/2018 | 308 NIS, 485 NPT, 523 NKT | 126, 143, 352, 353, 359 | |  | | 238 NIT, 359 NIS, 446 NNS, 508 NVT | 245, 246 |
| 137 | HPIV3/China_Ningxia/YC18068/2018 | 308 NIS, 485 NPT, 523 NKT | 126, 143, 352, 353, 359 | |  | | 238 NIT, 359 NIS, 446 NNS, 508 NVT | 245, 246 |
| 138 | HPIV3/China_Ningxia/YC18084/2018 | 308 NIS, 485 NPT, 523 NKT | 143, 352, 359 | |  | | 238 NIT, 359 NIS, 446 NNS, 508 NVT | 245, 246 |
| 139 | HPIV3/China_Ningxia/YC18085/2018 | 308 NIS, 485 NPT, 523 NKT | 126, 143, 352, 353, 359 | |  | | 238 NIT, 359 NIS, 446 NNS, 508 NVT | 245, 246 |
| 140 | HPIV3/China_Ningxia/YC19024/2019 | 308 NIS, 485 NPT, 523 NKT | 126, 143, 352, 353, 359 | |  | | 238 NIT, 359 NIS, 446 NNS, 508 NVT | 245, 246 |
| 141 | HPIV3/China_Ningxia/YC19075/2019 | 30 NKT, 308 NIS, 485 NPT, 523 NKT | 126, 143, 352, 353, 359 | |  | | 238 NIT, 359 NIS, 446 NNS, 508 NVT | 245, 246 |
| 142 | HPIV3/China_Zhejiang/WZ17121/2018 | 308 NIS, 485 NPT, 523 NKT | 126, 143, 352, 353, 359 | |  | | 238 NIT, 359 NIS, 446 NNS, 508 NVT | 245, 246 |
| 143 | HPIV3/China_Zhejiang/WZ17130/2018 | 308 NIS, 485 NPT, 523 NKT | 126, 143, 352, 353, 359 | |  | | 238 NIT, 359 NIS, 446 NNS, 508 NVT | 245, 246 |
| 144 | HPIV3/China_Zhejiang/WZ17143/2018 | 308 NIS, 485 NPT, 523 NKT | 126, 143, 352, 353, 359 | |  | | 238 NIT, 359 NIS, 446 NNS, 508 NVT | 245, 246 |
| 145 | HPIV3/China_Zhejiang/WZ17157/2018 | 308 NIS, 485 NPT, 523 NKT | 126, 143, 352, 353, 359 | |  | | 238 NIT, 359 NIS, 446 NNS, 508 NVT | 245, 246 |
| 146 | HPIV3/China_Zhejiang/WZ17159/2018 | 308 NIS, 485 NPT, 523 NKT | 126, 143, 352, 353, 359 | |  | | 238 NIT, 359 NIS, 446 NNS, 508 NVT | 245, 246 |
| 147 | HPIV3/China_Zhejiang/WZ17166/2018 | 308 NIS, 485 NPT, 523 NKT | 126, 143, 352, 353, 359 | |  | | 238 NIT, 359 NIS, 446 NNS, 508 NVT | 245, 246 |
| 148 | HPIV3/China_Zhejiang/WZ17167/2018 | 308 NIS, 485 NPT, 523 NKT | 126, 143, 352, 353, 359 | |  | | 238 NIT, 359 NIS, 446 NNS, 508 NVT | 245, 246 |
| 149 | HPIV3/China_Zhejiang/WZ17187/2018 | 308 NIS, 485 NPT, 523 NKT | 126, 143, 352, 353, 359 | |  | | 238 NIT, 359 NIS, 446 NNS, 508 NVT | 245, 246 |
| 150 | HPIV3/China_Zhejiang/WZ17193/2018 | 308 NIS, 485 NPT, 523 NKT | 143, 352, 353, 359 | |  | | 238 NIT, 359 NIS, 446 NNS, 508 NVT | 245, 246 |
| 151 | HPIV3/China_Zhejiang/WZ17195/2018 | 308 NIS, 485 NPT, 523 NKT | 126, 143, 352, 353, 359 | |  | | 238 NIT, 359 NIS, 446 NNS, 508 NVT | 245, 246 |
| 152 | HPIV3/China_Zhejiang/WZ17234/2018 | 308 NIS, 485 NPT, 523 NKT | 126, 143, 352, 353, 359 | |  | | 238 NIT, 359 NIS, 446 NNS, 508 NVT | 245, 246 |
| 153 | HPIV3/China_Zhejiang/WZ17275/2018 | 308 NIS, 485 NPT, 523 NKT | 126, 143, 352, 353, 359 | |  | | 238 NIT, 359 NIS, 446 NNS, 508 NVT | 245, 246 |
| 154 | HPIV3/China_Zhejiang/WZ17395/2019 | 308 NIS, 485 NPT, 523 NKT | 126, 143, 352, 353, 359 | |  | | 238 NIT, 359 NIS, 446 NNS, 508 NVT | 245, 246 |
| 155 | HPIV3/China_Zhejiang/WZ17443/2019 | 308 NIS, 485 NPT, 523 NKT | 126, 143, 352, 353, 359 | |  | | 238 NIT, 359 NIS, 446 NNS, 508 NVT | 245, 246 |
| 156 | HPIV4b/China Ningxia/YC17038/2017 | 279 NGT, 339 NST, 347 NKT, 502 NNS, 530 NLT | 340, 346, 350, 354, | |  | | 66 NYS, 74 NKT, 244 NIS | 439 |
